# Functional Characterization of the Effects of CsDGAT1 and CsDGAT2 on Fatty Acid Composition in *Camelina sativa*

**DOI:** 10.3390/ijms25136944

**Published:** 2024-06-25

**Authors:** Kyeong-Ryeol Lee, Yumi Yeo, Jihyea Lee, Semi Kim, Chorong Im, Inyoung Kim, Juho Lee, Seon-Kyeong Lee, Mi Chung Suh, Hyun Uk Kim

**Affiliations:** 1 Department of Agricultural Biotechnology, National Institute of Agricultural Sciences, Rural Development Administration, Jeonju 54875, Republic of Korea; 2 Department of Life Science, Sogang University, Seoul 04107, Republic of Korea; 3 Department of Bioindustry and Bioresource Engineering, Plant Engineering Research Institute, Sejong University, Seoul 05006, Republic of Korea

**Keywords:** CsDGAT1, CsDGAT2, CRISPR/Cas, triacylglycerol, camelina

## Abstract

Triacylglycerols (TAGs) are the storage oils of plant seeds, and these lipids provide energy for seed germination and valuable oils for human consumption. Three diacylglycerol acyltransferases (DGAT1, DGAT2, and DGAT3) and phospholipid:diacylglycerol acyltransferases participate in the biosynthesis of TAGs. DGAT1 and DGAT2 participate in the biosynthesis of TAGs through the endoplasmic reticulum (ER) pathway. In this study, we functionally characterized CsDGAT1 and CsDGAT2 from camelina (*Camelina sativa*). Green fluorescent protein-fused CsDGAT1 and CsDGAT2 localized to the ER when transiently expressed in *Nicotiana benthamiana* leaves. To generate *Csdgat1* and *Csdgat2* mutants using the CRISPR/Cas9 system, camelina was transformed with a binary vector carrying *Cas9* and the respective guide RNAs targeting *CsDGAT1s* and *CsDGAT2s* via the *Agrobacterium*-mediated floral dip method. The EDD1 lines had missense and nonsense mutations in the *CsDGAT1* homoeologs, suggesting that they retained some CsDGAT1 function, and their seeds showed decreased eicosaenoic acid (C20:1) contents and increased C18:3 contents compared to the wild type (WT). The EDD2 lines had a complete knockout of all *CsDGAT2* homoeologs and a slightly decreased C18:3 content compared to the WT. In conclusion, CsDGAT1 and CsDGAT2 have a small influence on the seed oil content and have an acyl preference for C20:1 and C18:3, respectively. This finding can be applied to develop oilseed plants containing high omega-3 fatty acids or high oleic acid.

## 1. Introduction

Seed oils with high energy density serve as a critical energy source for seed germination and seedling development. Seed oils are also extensively used by humans as food and for industrial purposes, including the production of biofuels and chemical feedstocks, the renewable and eco-friendly alternatives to fossil fuels, and feedstocks. Triacylglycerol (TAG), the storage lipid in seeds, is biosynthesized through sequential acylation with an acyl-CoA from glycerol-3-phosphate (G3P) by three acyltransferases, namely G3P acyltransferase (GPAT), lysophosphatidate acyltransferase (LPAT), and diacylglycerol acyltransferase (DGAT), in the Kennedy pathway [1]. Additionally, phosphatidylcholine:diacylglycerol acyltransferase (PDAT) biosynthesizes TAG in an acyl-CoA–independent manner [2,3]. Acylation of diacylglycerol (DAG) by DGAT is a rate-limiting step of seed oil accumulation in plant seeds [4].

Plants have three types of *DGAT*, all sharing the same function in TAG biosynthesis but differing in gene and protein structures. *DGAT1*, belonging to the membrane-bound *O*-acyltransferase (MBOAT) protein family, was initially identified in mice (*Mus musculus*) and later in various plants, including Arabidopsis (*Arabidopsis thaliana*) [5,6,7,8,9,10,11,12]. DGAT1 plays a critical role in seed oil accumulation in Arabidopsis [7,13]. In contrast, *DGAT2*, a member of the monoacylglycerol acyltransferase (MGAT) family, was initially discovered in the oleaginous fungus *Umbelopsis ramanniana* (formerly *Mortierella ramanniana*) [14]. *Arabidopsis thaliana DGAT2* (*AtDGAT2*), however, was found to have little impact on TAG biosynthesis, as the oilseed content was not changed in an Arabidopsis *dgat2* T-DNA insertional mutant [15]. Nevertheless, DGAT2s are involved in the accumulation of unusual fatty acids in plants that accumulate epoxy or hydroxy fatty acids in seed oil, such as the castor plant (*Ricinus communis*), ironweed (*Vernonia galamensis*), wild spurge (*Euphorbia lagascae*), and Stokes’ aster (*Stokesia laevis*) [16,17,18,19]. DGAT3, a soluble form of DGAT, was first found in peanut (*Arachis hypogaea*) cotyledons [20]. AtDGAT3 is involved in TAG accumulation in Arabidopsis and has a preference for α-linolenic acid (C18:3), as demonstrated using agroinfiltration in *Nicotiana benthamiana* leaves [21]. It was also reported that DGAT3 is involved in TAG biosynthesis in the immature seeds, flowers, and leaves of the tung tree (*Vernicia fordii*) [22].

Camelina (*Camelina sativa*), also called false flax or gold-of-pleasure, is an emerging oilseed crop in the Brassicaceae family [23,24,25]. Its seeds have a high oil content (35–45%) and a high C18:3 content (about 35%) compared to other common vegetable oils, and camelina seed oil is used to produce soaps, polishes, cosmetics, and biokerosene for use as aircraft fuel [23,24,26]. Its genome is allohexaploid (2*n* = 6*x* = 40), with three almost-identical homoeologous sets of chromosomes [27,28]. Camelina can be cultivated in poor soils due to its low nutrient requirements [23] and shows good tolerance of abiotic stresses such as drought and low temperatures [23,24]. Camelina also has a short life cycle (15–17 weeks), making it potentially amenable to double cropping and more economical to grow than other oilseed crops [26]. Furthermore, camelina can be easily transformed using the Agrobacterium (*Agrobacterium tumefaciens*)-mediated floral dip method [29,30], with a comparable transformation efficiency to Arabidopsis [31]. Therefore, camelina is considered an ideal host plant for vegetable oil biotechnology and industry [32].

Despite camelina’s long history of cultivation, unlike model plants, such as Arabidopsis, and major oilseed crops, such as rapeseed, few studies have explored the genes related to fatty acid metabolism, such as *DGAT*s, in camelina. The functional analysis of three camelina *DGAT1* genes (*CsDGAT1*) revealed that overexpressing *CsDGAT1B* in the Arabidopsis *dgat1* mutant (AS11) background increased the seed mass and storage oil content [11]. In in vitro assays of CsDGAT1 in microsomal fractions from developing seeds, artificial microRNA-mediated silencing of *CsDGAT1* increased the C18:3 content [33]. Lager et al. [34] showed that CsDGAT1 prefers acyl donors with saturated and monounsaturated fatty acids, while CsDGAT2 exhibits a high specificity for acyl acceptors containing only polyunsaturated fatty acids (PUFAs). Though DGAT3 has not been reported to have a major role in seed oil accumulation in plants, recent studies confirmed that CsDGAT3 is an important contributor to TAG accumulation in camelina seeds [35,36].

In this study, we investigated the roles of CsDGAT1 and CsDGAT2 in seed oil accumulation, as well as their fatty acid preferences, by generating *Csdgat1* and *Csdgat2* knockout (KO) camelina plants using the clustered regularly interspaced short palindromic repeats (CRISPR)/CRISPR-associated nuclease 9 (Cas9) system. CsDGAT1 and CsDGAT2 have a smaller impact on seed oil content than CsDGAT3 does, but their effects on TAG biosynthesis were sufficient to alter the fatty acid composition. The C18:3 content and the C20:1 content of the seed oil increased and decreased, respectively, in *Csdgat1* KO camelina plants, indicating that CsDGAT1 has a weak preference for C18:3 and a strong preference for C20:1, whereas the C18:3 content of seed oil decreased in *Csdgat2* KO camelina plants, indicating that CsDGAT2 has a strong preference for C18:3. These findings could potentially be applied for developing tailored oilseed plants.

## 2. Results

### 2.1. Phylogenetic Relationships of CsDGAT1s and CsDGAT2s

*CsDGAT1A*, *CsDGAT1B*, and *CsDGAT1C* were named as designated by Kim et al. [11] and are located on the chromosomes 19, 1, and 15, respectively, as revealed by a BLAST search in EnsemblPlants (Supplementary Table S1). *CsDGAT1A* and *CsDGAT1B* each comprise 1563 bp and encode the proteins of 520 amino acids (aa), similar to *Arabidopsis thaliana DGAT1* (*AtDGAT1*), whereas *CsDGAT1C* is 1566 bp long and encodes a protein of 521 aa. The similarities between *AtDGAT1* and *CsDGAT1*s are very high (93.6–93.8%).

The BLAST search results with *AtDGAT2* as a query in EnsemblPlants showed 90.1–90.4% similarities to *CsDGAT2*s. The three *CsDGAT2* homoeologs, each comprising 948 bp and encoding proteins of 315 aa, located on chromosomes 4 (Csa04g037310), 6 (Csa06g025650), and 9 (Csa09g058550), were named *CsDGAT2A*, *CsDGAT2B*, and *CsDGAT2C*, respectively. We analyzed the phylogenetic relationships among CsDGAT1s, CsDGAT2s, and 46 DGATs from various plant species and found that CsDGAT1, CsDGAT2, and CsDGAT3 clustered into different groups (Supplementary Figure S1). The three homoeologs of CsDGAT1 and CsDGAT2 were closely clustered with the DGAT1 and DGAT2 of Arabidopsis (and other *Brassica* spp.), respectively, but not with the DGATs from plant species belonging to different families, such as the peanut, soybean (*Glycine max*), and sesame (*Sesamum indicum*). These results suggest that the *DGAT1*, *DGAT2*, and *DGAT3* genes were independently diverged, but the three homoeologs of *CsDGAT1*, *CsDGAT2*, and *CsDGAT3* have been diverged from the common ancestor of camelina species [27].

Although all plant DGATs function in TAG biosynthesis, they share a low identity with each other. Plant DGAT1s and DGAT2s have 14–16 and 8–9 exons, respectively [20,21,37]. In addition, DGAT1 and DGAT2 have 6–9 and 1–2 transmembrane domains (TMDs), respectively [37]. The UniProt analysis revealed that CsDGAT1s have 16 exons and 9 TMDs, while CsDGAT2s have 9 exons and 2 TMDs (Supplementary Table S1, Figure S2). RARESPLSSDAIFKQSHAG, just upstream of TMD1 in CsDGAT1, was assumed to be a putative acyl-CoA-binding site [38]. Another proposed acyl-CoA-binding site, FYXDWWN, which is largely conserved among DGAT1s, was found adjacent to TMD6 [39,40]. The putative DAG/phorbol ester binding site, HKWXXRHXYXP, was also located between TMD6 and TMD7 [7]. We found similar putative active motifs of acyltransferases, comprising a conserved histidine and aspartic acid residue (HXXXXD and HXXXD), adjacent to TMD4 and TMD5 in CsDGAT1 [37]. DGAT2 has not been previously reported to possess a putative acyl-CoA-binding site or a putative DAG-binding site, nor did we identify one; however, we found a putative acyltransferase active motif, HXXXD, from aa 185 to 189, in the CsDGAT2 homoeologs.

### 2.2. Both DGAT1 and DGAT2 Are Membrane-Bound Proteins Localized to the Endoplasmic Reticulum (ER)

**Figure 1:**
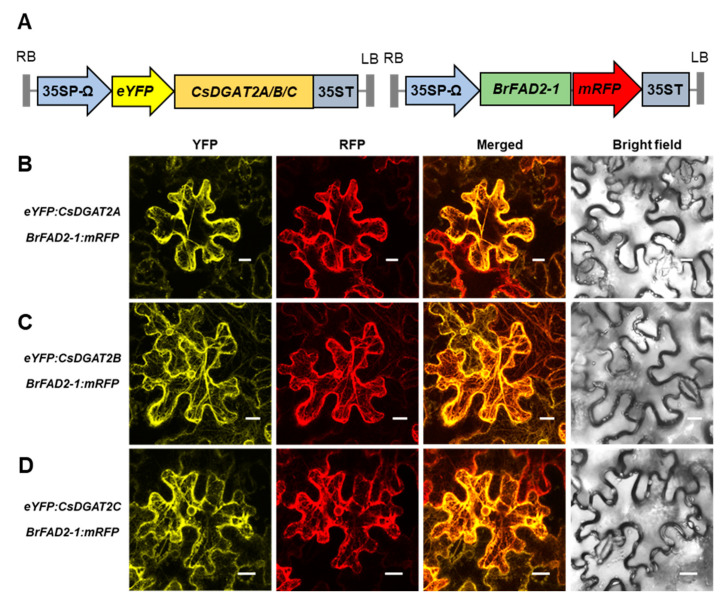
Subcellular localization of CsDGAT2A, CsDGAT2B, and CsDGAT2C in *N. benthamiana* leaf epidermal cells. (**A**) Binary vector construct for *eYFP*:*CsDGAT2*s and *BrFAD2-1*:*mRFP*. 35SP-Ω, cauliflower mosaic virus 35S promoter with 5′ leader sequence (Ω) of tobacco mosaic virus; LB, left border; RB, right border; 35ST, 35S terminator; YFP, yellow fluorescent protein; mRFP, monomeric red fluorescent protein. (**B**–**D**) Agrobacterium harboring one of the *eYFP*:*CsDGAT2*s and the *BrFAD2-1*:*mRFP* construct was infiltrated into *N. benthamiana* leaves, and then the yellow and red fluorescence signals were visualized under laser confocal scanning microscopy. Bars, 20 μm.

DGAT1 and DGAT2 are endoplasmic reticulum (ER) membrane-bound proteins [17]. The ER retrieval motif of CsDGAT1 is YYHDL (Supplementary Figure S2A), similar to those in Arabidopsis and tung tree VfDGAT1s [17]. The ER retrieval motif of CsDGAT2 is LQLNI (Supplementary Figure S2B), which differs from the LELKI motif of AtDGAT2 and the LKLEI motif of VfDGAT2 [17,41]. Previously, agroinfiltration in *Nicotiana benthamiana* leaf epidermal cells using constructs expressing *enhanced yellow fluorescent protein* (*eYFP*)-fused *CsDGAT1*s revealed that the CsDGAT1s were localized to the ER [11]. To analyze the subcellular localization of CsDGAT2s, we co-infiltrated Agrobacterium carrying e*YFP*:*CsDGAT2*, *Brassica rapa BrFAD2-1*:*mRFP* (an ER marker), and tobacco bushy stunt virus p19 as a gene-silencing suppressor into 5-week-old *N. benthamiana* leaves. At 48 h after infiltration, we observed eYFP and mRFP fluorescence signals in the ER, indicating that all CsDGAT2s localize to the ER (Figure 1). Quantification of the co-localized signal intensity in the fluorescence images using Pearson’s correlation coefficient supported the localization of all three CsDGAT2s to the ER (Supplementary Figure S3).

### 2.3. CsDGAT1 and CsDGAT2 Have Seed-Specific Expression, but the Expression Level of CsDGAT1 Was Higher

To determine the expression pattern and the transcript level for each homoeolog of *CsDGAT1* and *CsDGAT2* in camelina, we cloned all homoeologs and designed homoeolog-specific primers. However, despite using homoeolog-specific primers, we were unable to obtain homoeolog-specific amplification. Since camelina has more than 97% identity among homoeologs, designing efficient homoeolog-specific primers is difficult. According to the method of Hayashi et al. [42], we attempted to confirm homoeolog-specific amplification using primers in which one or more mismatch sequences were replaced at the 3′ end region, but the expression level was too low. Even after several trials using different 3′-end mismatch sequences, the homoeolog-specific reverse-transcription quantitative PCR (RT-qPCR) was unsuccessful. Therefore, we measured the total transcript level for each set of three homoeologs, but not that of the individual homoeologs. The transcript levels of the *CsDGAT1*, *CsDGAT2*, *CsDGAT3*, and *CsPDAT1* genes in flowers, leaves, stems, roots, cotyledons, and developing seeds at stage 1 (DS1), DS2, DS3, DS4, and DS5 are shown in Supplementary Figure S4. The *CsDGAT1*s, *CsDGAT2*s, and *CsDGAT3*s showed seed-specific expression, and their expression pattern in developing seeds showed a bell-shaped pattern similar to that of the genes related to seed oil biosynthesis and accumulation (Supplementary Figure S4A–C). In developing seeds, the highest expression levels of *CsDGAT1* was 6.5-fold higher than that of *CsDGAT2* but was only 32% that of *CsDGAT3*. *CsDGAT3* expression was much higher than that of the other genes in all tissues. *CsDGAT2* expression peaked at DS3, which differed from the expression of *CsDGAT1* and *CsDGAT3*, which peaked at DS4 (Supplementary Figure S4A–C). These results were similar to those reported previously [36]. In contrast to the seed-specific expression of *CsDGAT*s, the expression level of *CsPDAT1*s, which are also involved in seed oil biosynthesis, was highest in leaves and cotyledons, and the expression of *CsPDAT1*s in developing seeds did not show a bell-shaped pattern similar to those of other genes related to seed oil biosynthesis and accumulation (Supplementary Figure S4D). Taken together, these results indicate that *CsDGAT1*s, *CsDGAT2*s, and *CsDGAT3*s are expressed mainly in developing seeds, and the expression level of *CsDGAT1*s is higher than that of *CsDGAT2*s, but lower than that of *CsDGAT3*s.

### 2.4. Csdgat1 KO Lines and Csdgat2 KO Lines Were Obtained Using CRISPR/Cas

To generate *CsDGAT1* and *CsDGAT2* KO camelina plants, we used the pHEE401E-Red vector, which can accommodate the insertion of two guide RNAs [36]. We selected two optimal gRNA sequences to knock out all *CsDGAT1* and *CsDGAT2* homoeologs, respectively, using Cas Designer [43] according to the criteria described in Section 4 (Supplementary Table S2). The dual gRNAs for *CsDGAT1*s or *CsDGAT2*s were inserted into pHEE401E-Red, and the complete vectors were named pRedEDD1 (EC1.2P:*zCas9* + Dual gRNA for *DGAT1*) and pRedEDD2, respectively (Supplementary Figure S5). Camelina plants were transformed using the floral dip method with the Agrobacterium strain GV3101, carrying pRedEDD1 or pRedEDD2. We selected 67 and 172 T_1_ camelina seeds for EDD1 and EDD2, respectively, emitting orange–red fluorescence (Supplementary Figure S6A,B). The transformation efficiencies of EDD1 and EDD2 camelina plants were approximately 0.25% and 0.37%, respectively (Supplementary Table S3).

To determine the insertion–deletion mutant (indel) frequency of transformed camelina, we conducted homoeolog-specific PCR followed by Sanger sequencing. First, we designed *CsDGAT1* and *CsDGAT2* homoeolog-specific primers to determine the indel frequency, and the sequencing results of the PCR products using WT genomic DNA demonstrated that they amplified the individual homoeologs. Next, we performed homoeolog-specific PCR using the EDD1 and EDD2 plants (Supplementary Figure S6C,D), followed by Sanger sequencing of the PCR products. Finally, we analyzed the homoeolog-specific PCR-based Sanger sequencing results using two web-based tools, TIDE and DsDecodeM [44,45]. Our results showed that in the 67 T_1_ EDD1 lines, only five individuals had over 15% indel frequency in a single homoeolog (Supplementary Table S4). Only EDD1#22 had over 15% indel frequency in all three homoeologs of *CsDGAT1*, whereas EDD1#10, #45, #47, and #49 had over 15% indel frequency in one or two homoeologs of *CsDGAT1*. To obtain a complete *Csdgat1* KO camelina line, we selected five EDD1 T_1_ lines for T_2_ generation by self-fertilization. In the 172 T_1_ EDD2 lines, 34 individuals had over 15% indel frequency in a single homoeolog (Supplementary Table S5). Compared to the EDD1 lines, there were many more gene-edited EDD2 lines (8.5% vs. 21.1%), and the proportion of lines with over 15% indel frequency in all three homoeologs was much higher (20.0% vs. 58.8%). In particular, EDD2#25 and EDD2#66 were predicted to be complete *CsDGAT2* KO lines. Therefore, we chose the 12 EDD2 T_1_ lines (EDD2#9, #24, #25, #26, #42, #44, #59, #66, #83, #106, #137, and #147) with high indel frequencies in all *CsDGAT2* homoeologs as the T_2_ generation.

The indel patterns and zygosities of EDD1 and EDD2 T_2_ lines, except for the lines that were confirmed to be unedited at all, are listed in Table 1 and Table 2. Deletions of 120–121 nt and 36–41 nt, spanning the sgRNA1 and sgRNA2 sites, were found in EDD1 and EDD2 lines, respectively. Interestingly, cases of co-occurrence of indels at two sgRNA sites on the same haploid gene were identified in the T_2_ descendants of EDD1#10 and #22 and those of EDD2#25 and #137 (Table 1 and Table 2). However, all of these cases were expected to result in missense mutations. Overall, there were a large number of indel patterns that were expected to cause missense mutations. In the EDD1 T_2_ generation, we did not find a complete *Csdgat1* KO line. EDD1#22-1 and #22-4, which had a missense mutation in only the haploid of *CsDGAT1A* and nonsense mutations in the other *CsDGAT*s (aa′bbcc; lowercase letters and ′ indicate a nonsense mutation and a missense mutation, respectively), were expected to have the lowest CsDGAT1 activity among the EDD1 T_2_ descendants (Table 2). In the EDD2 T_2_ generation, complete *Csdgat2* KO lines were found in the T_2_ descendants of EDD2 #42, #59, and #66 (Table 2).

### 2.5. EDD1 and EDD2 Seeds Have Changed Fatty Acid Content Compared to WT Seeds

**Figure 2:**
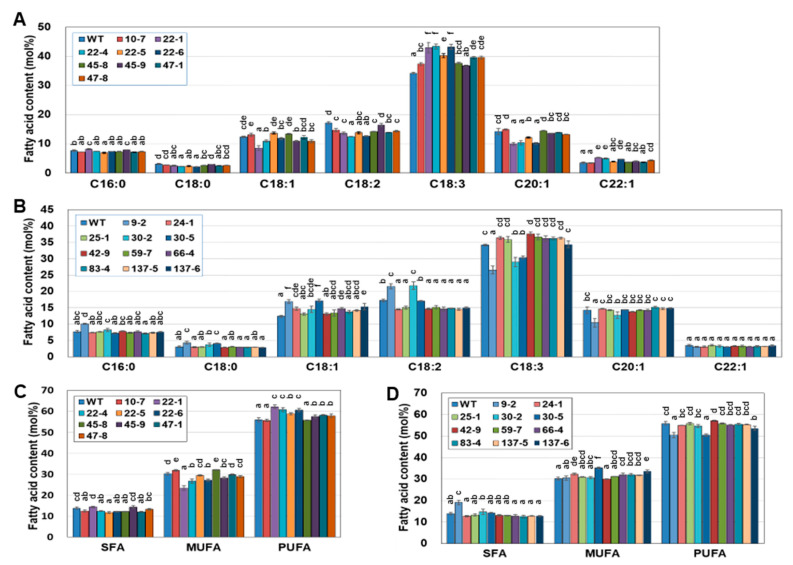
The composition of each fatty acid and fatty acid type from EDD1 and EDD2 T_2_ seeds. (**A**) Seed fatty acid composition of EDD1 mutants. (**B**) Saturated fatty acid (SFA), monounsaturated fatty acid (MUFA), and polyunsaturated fatty acid (PUFA) content of EDD1 mutant seeds. (**C**) Seed fatty acid composition of EDD2 mutants. (**D**) SFA, MUFA, and PUFA content of EDD2 mutant seeds. In (**A**,**B**), the minor fatty acids such as C20:0 and C20:2 were omitted. All experiments were performed in triplicate and the error bar is standard error. The values were statistically analyzed using one-way ANOVA followed by Tukey’s test (*p* < 0.05). Lowercase letters above the bars indicate the results of a one-way ANOVA statistical test.

To observe how the mutations in *CsDGAT1* and *CsDGAT2* caused changes in the fatty acid composition of seeds, we analyzed the seed fatty acids from EDD1 and EDD2 T_2_ lines. Nine EDD1 T_2_ lines and 11 EDD2 T_2_ lines were used for seed fatty acid analysis. EDD1#10-7, #22-1, #22-4, #22-5, #22-6, #45-8, #45-9, #47-1, and #47-8; EDD2#9-2, #24-1, #25-1, #30-2, #30-5, #42-9, #59-7, #66-4, #83-4, #137-5, and #137-6. The changes in seed fatty acid composition of the EDD1 T_2_ plants are shown in Figure 2A. The C16:0 contents of EDD1 T_2_ plants were generally similar to those of the WT. The C18:2 contents of the EDD1 T_2_ lines were generally lower than that of the WT, while the C18:1 and C20:1 contents of some EDD1 T_2_ lines were lower than those of the WT. By contrast, the C18:3 contents of most EDD1 T_2_ lines were increased compared to that of the WT. EDD1#45-9 was an exception, in that the C18:2 and C18:3 contents were not significantly changed. Among the EDD1 T_2_ lines, #22-4 had a C18:2 content of 12.4 mol%, which was a decrease of 4.8 mol% compared to WT, and a C18:3 content of 43.4 mol%, an increase of 9.2 mol% compared to WT. The C20:1 and erucic acid (C22:1) contents of EDD1 T_2_ lines were similar to those of the WT, except for the T_2_ descendants of #22 (#22-1, -4, -5, and -6), which generally showed a decrease in the C20:1 content and an increase in the C22:1 content.

Figure 2C showed the comparison results by grouping saturated fatty acids (SFAs), monounsaturated fatty acids (MUFAs), and PUFAs. In the seed oil from EDD1 T_2_ plants, the SFA contents were significantly decreased compared to the WT, except for #10-7, #45-9, and #47-8. Compared to the MUFA content of the WT, that of #47-1 and #47-8 was similar, whereas that of #10-7 and #45-8 was significantly increased, and that of other lines was significantly decreased. Compared to the PUFA content of the WT, those of all EDD1 lines were increased, except for #10-7 and #45-8. However, most of the individuals with a significantly increased C18:3 content had a decreased C18:2 content (Figure 2A); thus, the PUFA content of all EDD1 lines slightly increased compared to that of the WT. EDD1 #22-1 had a PUFA content increased by 6.3 mol% and a MUFA content decreased by 6.8 mol% compared to the WT, showing the greatest fatty acid changes.

The changes in the seed fatty acid composition of the EDD2 T_2_ lines are shown in Figure 2B. Compared to the palmitic acid (C16:0) and stearic acid (C18:0) contents of the WT, only #9-2 increased. The C18:1 content was similar or increased compared to the WT, and the increase in #9-2 and #30-5 was remarkable among the EDD2 T_2_ lines. Compared to the WT, the C18:2 content was increased in #9-2 and #30-2, similar in #30-5, and decreased in the other lines. The C18:3 content was decreased in #9-2, #30-2, and #30-5 and significantly increased only in #42-9; the rest were similar to the WT. The C20:1 content decreased only in #9-2, and the rest were similar to the WT. The C22:1 content of EDD2 T_2_ lines was almost the same as that of the WT. Figure 2D shows the SFA, MUFA, and PUFA contents of seed oil from EDD2 T_2_ plants. The SFA content of seed oil from EDD2 T_2_ plants increased only in #9-2 compared to that of the WT. The MUFA content was increased in #24-1, #30-3, and #137-6, while the rest were similar to that of the WT. The PUFA contents were all similar, except in #9-2 and #137-6, in which they were decreased compared to the WT. Overall, the seed fatty acid composition of EDD1 T_2_ lines differed significantly among individuals, whereas that of EDD2 T_2_ lines was relatively similar in all individuals, except for #9-2, #30-2, and #30-5, which changed dramatically compared to the WT. Taken together, the seed fatty acid composition of the EDD1 T_2_ lines was different from that of the WT and slightly differed among each other. In contrast, the seed fatty acid composition of the EDD2 T_2_ lines, except for the #9-2, #30-2, and #30-5 lines, which demonstrated dramatic changes, showed an insignificant difference from that of the WT.

**Figure 3:**
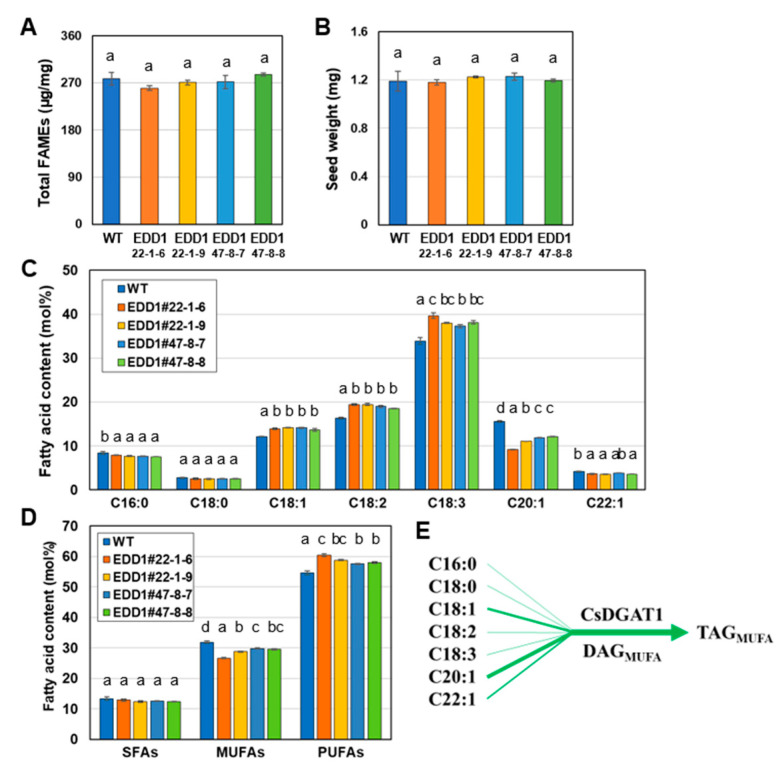
Seed phenotype analysis of T_3_ EDD1 mutants. (**A**) Total FAMEs per mg seeds. (**B**) Seed weight. (**C**) Seed fatty acid content. (**D**) Fatty acid content of each of the three different types: saturated fatty acid (SFA), monounsaturated fatty acid (MUFA), and polyunsaturated fatty acid (PUFA). (**E**) Biosynthetic pathway of TAG (triacylglycerol) from DAG (diacylglycerol) and C16:0 to C22:1 fatty acids by CsDGAT1. This indicates that CsDGAT1 prefers DAGs with MUFAs and MUFAs such as C18:1 and C20:1 as the acyl substrates, resulting in the biosynthesis of TAGs with a high MUFA content. In (**C**), minor fatty acids such as C20:0 and C20:2 were omitted. All experiments were performed in triplicate, and the error bar is the standard error. The values were statistically analyzed using one-way ANOVA followed by Tukey’s test (*p* < 0.05). Lowercase letters above the bars indicate the results of a one-way ANOVA statistical test.

Next, we investigated seed fatty acid composition, seed oil content, and seed weight in #22-1-6 (aabbcc), #22-1-9 (a′a′bbcc), and #47-8-7 and #47-8-8 (aabbc′c′) for the EDD1 T_3_ lines (Figure 3). The seed oil content and seed weights of EDD1 T_3_ lines were similar to those of the WT (Figure 3A,B). The seed fatty acid composition of the EDD1 T_3_ lines showed an increase in C18:1, C18:2, and C18:3 contents, and a decrease in C16:0 and C20:1 contents compared to those of the WT (Figure 3C). In particular, #22-1-6 had the highest C18:1 content and the lowest C20:1 content among the EDD1 T_3_ lines. This may be because #22-1-6 (aabbcc) is a complete *Csdgat1* KO line, different from the other EDD1 T_3_ lines (a′a′bbcc and aabbc′c′). Comparing Figure 2A and Figure 3C, an increased C18:3 content and a decreased C20:1 content were similar between the EDD1 T_2_ and T_3_ lines. The C18:2 and C18:3 contents were decreased in the EDD1 T_2_ lines but increased in the EDD1 T_3_ lines. Although we noted some differences in seed fatty acid composition between the EDD1 T_2_ and T_3_ lines compared to the WT, the contents of SFAs, MUFAs, and PUFAs were similar between the EDD1 T_2_ and T_3_ lines compared to those of the WT (Figure 2C and Figure 3D). MUFA and PUFA contents in both EDD1 T_2_ and T_3_ lines were decreased and increased, respectively, compared to those of WT. Overall, this suggests that CsDGAT1 contributes less than CsDGAT3 to seed oil accumulation in camelina seeds [36], and CsDGAT1 has a strong preference for C20:1 but a weak preference for C18:3, resulting in the TAG with a high MUFA content (Figure 3E). However, this means that CsDGAT1 contributes a certain degree in TAG biosynthesis because the DGATs share functional redundancy in TAG biosynthesis.

**Figure 4:**
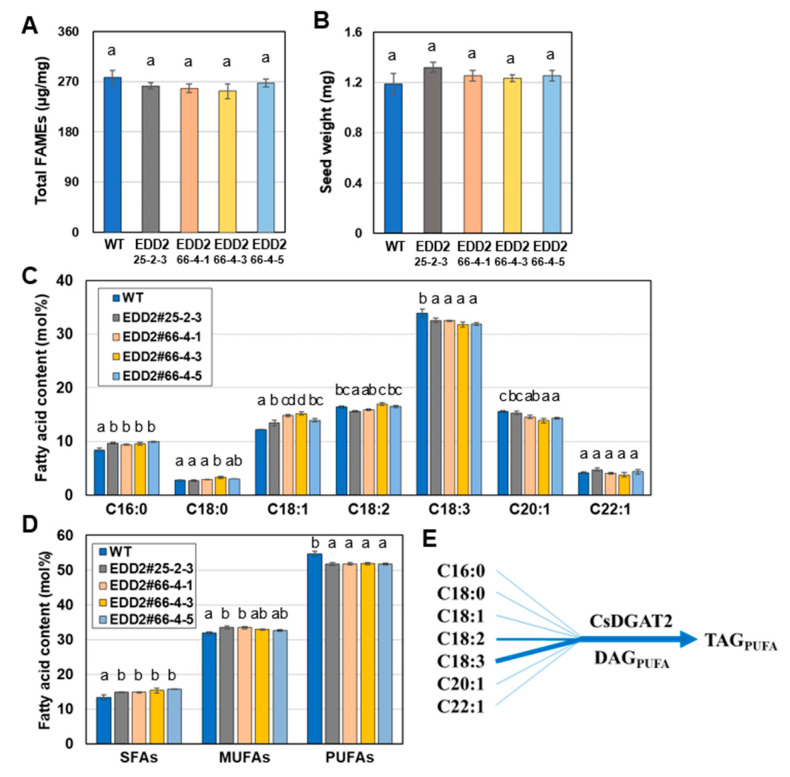
Seed phenotype analysis of T_3_ EDD2 mutants. (**A**) Total FAMEs per mg seeds. (**B**) Seed weight. (**C**) Seed fatty acid content. (**D**) Fatty acid content of each of the three different types: saturated fatty acid (SFA), monounsaturated fatty acid (MUFA), and polyunsaturated fatty acid (PUFA). (**E**) Biosynthetic pathway of TAG (triacylglycerol) from DAG (diacylglycerol) and C16:0 to C22:1 fatty acids by CsDGAT2. This indicates that CsDGAT2 prefers DAGs with PUFAs and PUFAs, especially C18:3, as the acyl substrates, resulting in the biosynthesis of TAGs with a high PUFA content. In (**C**), minor fatty acids such as C20:0 and C20:2 were omitted. All experiments were performed in triplicate, and the error bar is the standard error. The values were statistically analyzed using one-way ANOVA followed by Tukey’s test (*p* < 0.05). Lowercase letters above the bars indicate the results of a one-way ANOVA statistical test.

As shown in Figure 2B, EDD2#9-2, #30-2, and #30-5 had a lower C18:3 content than other EDD2 T_2_ lines and the WT. However, they also had a lower seed weight and an abnormal seed morphology, with seeds that were flat, curved, twisted, and tiny compared to those of other EDD2 lines and WT (Supplementary Figure S7). The cause of this reduced seed oil content and abnormal seed morphology was difficult to determine. Therefore, we excluded them from our investigation of seed phenotype in the T_3_ generation and assessed the seed fatty acid composition, seed oil content, and seed weight in the EDD2 T_3_ lines #25-2-3 (a′a′bbcc), #66-4-1, #66-4-3, and #66-4-5 (aabbcc) (Figure 4). These EDD2 T_3_ lines had seed oil contents similar to those of the WT (Figure 4A) and seed weights slightly but non-significantly higher than those of the WT (Figure 4B). The seed fatty acid composition showed a slight difference between the EDD2 T_3_ lines and the WT: both C16:0 and C18:1 contents were higher in the EDD2 T_3_ lines, while the C18:3 content was lower (by up to 1.9 mol%). The C18:2 and C20:1 contents showed a slight but statistically significant decrease in some lines (Figure 4C). The SFA and MUFA contents of EDD2 T_3_ lines were slightly higher, and the PUFA contents were lower than those in the WT (Figure 4D). They also indicate that CsDGAT2s have a strong preference for C18:3, resulting in a TAG with high PUFA content (Figure 4E). Together, these results suggest that CsDGAT2s are less crucial than CsDGAT3s for seed oil accumulation in camelina [36], but still contribute somewhat to the fatty acid composition.

### 2.6. EDD1 and EDD2 Lines Were Phenotypically Similar to WT

**Figure 5:**
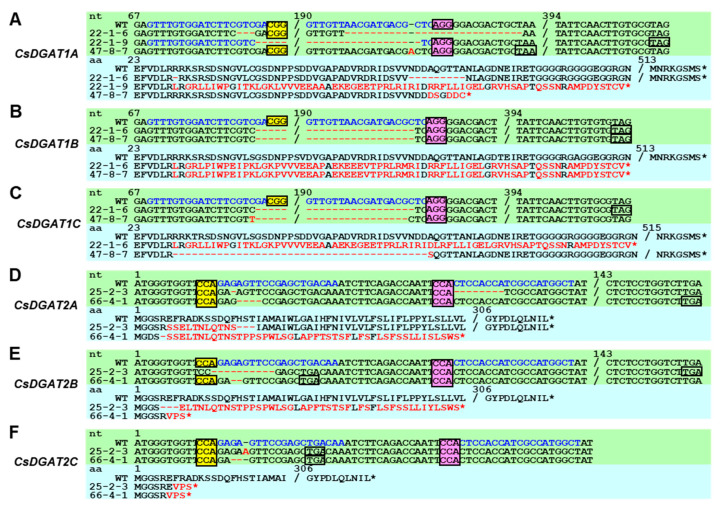
Nucleotide sequences (pale-green background) and deduced amino acid sequences (sky-blue background) of *CsDGAT1* and *CsDGAT2* homoeologs from T_2_ mutants. (**A**–**C**) *CsDGAT1A* to *CsDGAT1C* homoeologs from EDD1 mutants and (**D**–**F**) *CsDGAT2A* to *CsDGAT2C* homoeologs from EDD2 mutants showed diverse mutation types. The indel patterns of *CsDGAT1B* and *CsDGAT1C* in EDD1#22-1-9 were identical to those in #22-1-6. Red letters indicate different nucleotides or amino acids compared to WT. Red dashes and asterisks represent gaps produced by deletions and the termination of translation, respectively. Blue nucleotide sequences are sgRNA1 and sgRNA2. Yellow box and pink box indicate the protospacer-adjacent motif (PAM) sequence of sgRNA1 and sgRNA2, respectively, and the blank box indicates the premature stop codon caused by deletion. Asterisks indicate the termination of the translation by a stop codon.

Figure 5 shows the nucleotide and deduced amino acid changes of *CsDGAT1*s and *CsDGAT2*s from the EDD1 and EDD2 lines. For example, 3-nt and 27-nt deletions at each sgRNA target site in the *CsDGAT1A* of EDD1#22-1-6 caused 1-aa and 9-aa deletions, respectively. Meanwhile, a 121-nt deletion in the *CsDGAT1A* of EDD1#22-1-9 and a 1-nt insertion in the *CsDGAT1A* of #47-8-7 resulted in the early termination of translation at the 100-aa and 75-aa positions, respectively (Figure 5A). The *CsDGAT1B* of EDD1#22-1-6, #22-1-9, and #47-8 had a 121-nt deletion at the target site from sgRNA1 to sgRNA2, which resulted in the early termination of translation with altered amino acid residues at the 100-aa position (Figure 5B). The *CsDGAT1C* of EDD1#22-1-6 had the same 121-nt deletion as *CsDGAT1B* in the three lines as described above, resulting in the premature termination of translation. The *CsDGAT1C* of #47-8-7 had a 120-nt deletion, resulting in a 40-aa-deletion in CsDGAT1C (Figure 5C). In *CsDGAT2A*, EDD2#25-2-3 had 1-nt+8-nt deletion at each sgRNA site within a haploid, resulting in a 3-aa deletion and a change in 11 aa compared to the WT, but not early termination. EDD2#66-4-1 had a 4-nt deletion, resulting in early translation termination with aberrant amino acid residues (Figure 5D). The proteins encoded by *CsDGAT2B* in EDD2#25-2-3 and #66-4-1 were truncated due to a 10-nt deletion and a 2-nt deletion, respectively (Figure 5E). The *CsDGAT2C* of EDD2#25-2-3 and #66-4-1 had a 1-nt insertion and a 2-nt deletion, respectively (Figure 5F). The changes in the nucleotide sequences of *CsDGAT1*s and *CsDGAT2*s were also shown by Sanger sequencing (Supplementary Figures S8 and S9).

**Figure 6:**
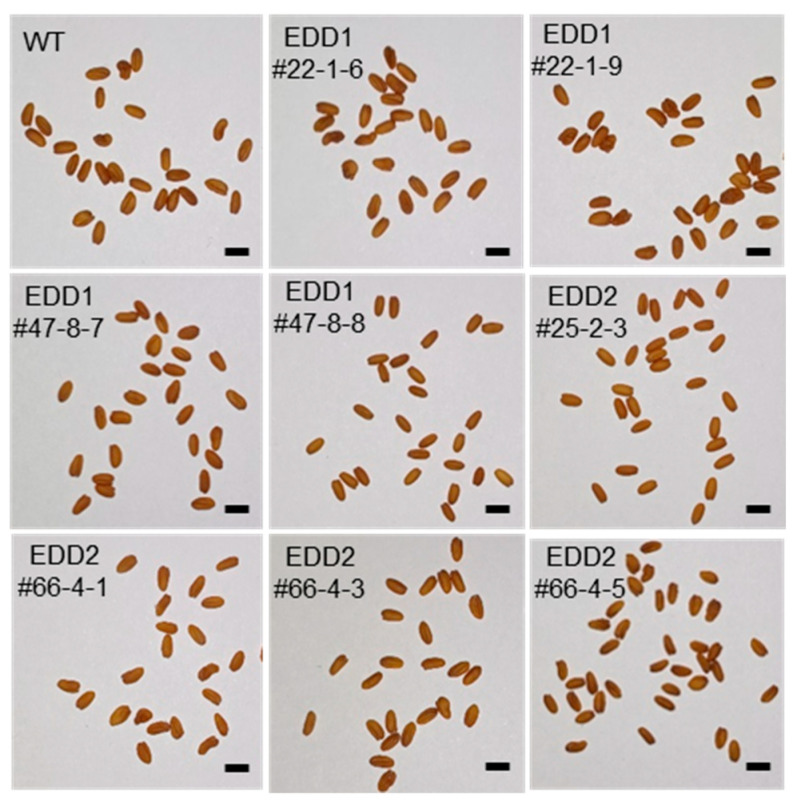
Seed morphology of EDD1 and EDD2 T_3_ lines. Scale bar, 2 mm.

We investigated other seed phenotypes, such as seed morphology and germination rate, in the same lines analyzed in Figure 3 and Figure 4: EDD1#22-1-6, #22-1-9, #47-8-7, and #47-8-8; and EDD2#25-2-3, #66-4-1, #66-4-3, and #66-4-5. Their seed morphology was normal, similar to that of the WT (Figure 6). Some abnormal seeds were observed in the EDD1 and EDD2 lines, and even in the WT, but most seeds were normal. We observed whether there was any change in seed germination rate due to the KO of *CsDGAT1* or *CsDGAT2*. Interestingly, all EDD1 and EDD2 lines, even EDD3#1-5-7 lines with about 19% decreased seed oil content [36], showed a 100% germination rate at 1 day after imbibition (DAI), identical to the WT. Thus, even though the functions of *CsDGAT1* and *CsDGAT2* were disrupted in the EDD1 and EDD2 lines, respectively, this did not affect their germination rates.

## 3. Discussion

As mentioned above, excellent agronomic traits such as cold tolerance, drought tolerance, low fertilizer requirements, and a short crop cycle make camelina an emerging and promising oilseed crop for industrial applications [24], whose triacylglycerol biosynthetic pathway and genes involved in seed oil accumulation have been gradually unveiled. Although Arabidopsis and camelina are closely related species belonging to the Brassicaceae family, the expression levels of the *DGAT* genes differ. DGAT1 is the main acyltransferase contributing to the seed oil content in Arabidopsis [7]. However, we previously showed that in camelina, the expression level of *DGAT3* is the highest among the *DGAT*s, and the *Csdgat3* mutant camelina has reduced seed oil content, suggesting that CsDGAT3 is mainly involved in seed oil accumulation, more so than the other CsDGATs in this species [36]. Therefore, we wondered to what extent CsDGAT1 and CsDGAT2 are involved in seed oil accumulation, as well as which fatty acids CsDGAT1 and CsDGAT2 prefer during TAG biosynthesis. In this study, we determined that (1) *CsDGAT1* and *CsDGAT2* in camelina are expressed in a seed-specific manner, but have lower expression levels compared to *CsDGAT3*; (2) the three CsDGAT2 proteins localize to the ER; (3) the levels of seed oil in EDD1 and EDD2 lines were similar to those of the WT; (4) EDD1 lines had higher C18:3 and lower C20:1 contents in seed oil than in the WT; and (5) the seed fatty acid composition in the EDD2 lines was slightly reduced in the C18:3 content compared to that in the WT. These results indicate that CsDGAT1 and CsDGAT2 contribute less to seed oil content than CsDGAT3, but have sufficient activity to alter the seed fatty acid composition. Although these results suggest that CsDGAT1 and CsDGAT2 make only minor contributions to seed oil content, their contributions were sufficiently high that the seed oil content in *Csdgat3* KO camelina plants was reduced by only 18.8% because of the functional redundancy of the three DGATs and PDATs involved in seed oil biosynthesis [36]. Therefore, CsDGAT1 and CsDGAT2 play minor but appropriate roles in seed oil biosynthesis. These findings show that the activity of seed oil-forming DGATs in camelina differs from that in Arabidopsis.

It is known that both DGAT1 and DGAT2 are ER-membrane-bound proteins and have the function of the TAG biosynthesis in the ER. CsDGAT1s have already been confirmed to be localized in the ER [11]. Therefore, in this study, it was important to determine whether CsDGAT2s were also localized in the ER. As shown in Figure 1, CsDGAT2s were confirmed to be localized and retained in the ER. According to McCartney et al. [46], the ER retrieval motif of plant ER membrane-bound proteins has a hydrophobic amino acid residue at the first and fifth positions and a K, R, D, or E residue at the fourth position (Φ-X-X-K/R/D/E-Φ, Φ hydrophobic amino acid residue). The ER retrieval motif of CsDGAT1 is YYHDL, similar to that of AtDGAT1 (Supplementary Figure S2A), and the ER retrieval motif of AtDGAT2 and VfDGAT2 is consistent with the LELKI consensus sequence [37]. In contrast, upon examining the amino acid sequences of DGAT2 of numerous plant species, we found that most of the fourth amino acid residues were K/R/E, but some were L/V. As in the case of CsDGAT2, the ER retrieval motif of *Brassica napus* BnDGAT2a and BnDGAT2 was LQLNI, which did not match the consensus motif [10,37]. McCartney et al. [46] showed that AtFAD2 with K/R/D/E at the fourth residue of the ER retrieval motif localized to the ER, but FAD2 with A or Q as the fourth residue did not. This suggests that having K/R/D/E as the fourth residue may not be an absolute requirement for the ER retrieval motif and that other, unknown factors affect the ER retention function of the ER retrieval motif.

In this study, we did not measure the transcript levels of each homoeolog of *CsDGAT1* and *CsDGAT2*, but rather the overall transcript levels of *CsDGAT1*s and *CsDGAT2*s. To obtain amplifications specific to homoeologs with very high identity (>97%), it is thought that it would be helpful to design primers specific to regions of low identity, such as the 3′-UTR, or to combine the methods used by Hayashi et al. [42]. The expression levels of *CsDGAT1* and *CsDGAT2* were significantly lower than that of *CsDGAT3* (Supplementary Figure S4A–C). This implies that while CsDGAT3 plays a critical role in determining seed oil content [36], the contribution of CsDGAT1 and CsDGAT2 to TAG biosynthesis might be lower than that that of CsDGAT3. Indeed, the seed oil content was not lower in the EDD1 lines than in the WT (Figure 3A), suggesting that CsDGAT1 has only a minor effect on seed oil biosynthesis, and the loss of its function can be compensated for by other CsDGATs. In Arabidopsis, *AtDGAT1* is the most dominant *DGAT* and the *dgat1* mutant (AS11) has a 25% reduced seed oil content [7,13]. Meanwhile, in camelina, *CsDGAT3* is dominant [36]. At its highest expression level, *CsDGAT2* expression is only 15% that of *CsDGAT1* and 5% that of *CsDGAT3* (Supplementary Figure S4B). Therefore, CsDGAT2 was predicted to have a smaller effect on seed oil biosynthesis than other CsDGATs. As expected, the seed oil content of the EDD2 lines did not differ from that of the WT (Figure 4A).

In Arabidopsis, AtDGAT1 has an acyl preference mainly for C18:1 but little preference for C18:3, whereas AtDGAT2 showed a preference for C18:1, C18:2, and C18:3 [41]. In camelina, CsDGAT1 showed a similar preference for all fatty acids constituting seed oil; in contrast, CsDGAT2 showed a much higher specificity for C18:3 than for other fatty acids in yeast heterologously expressing *CsDGAT1* or *CsDGAT2* [34]. However, it does not seem to occur in plant definitely because di-6:0-DAG, which does not occur naturally in plant seeds, was used as an acyl acceptor. When diverse acyl acceptors and C18 unsaturated acyl-CoAs (C18:1-, C18:2-, and C18:3-CoAs) were provided, CsDGAT1 preferred DAGs with relatively low degrees of unsaturation (di-C18:1-DAG and C16:0/C18:2-DAG) as the acyl acceptors and preferred all acyl-CoAs except when the acyl acceptors provided were di-C18:1-DAG and C18:1-CoA. Under the same condition, CsDGAT2 preferred DAGs with high degrees of unsaturation (di-C18:2-DAG and di-C18:3-DAG) as the acyl acceptors, with strongest preference for C18:3, followed by C18:2 [34]. CsDGAT1 may generate TAGs with 4 or 5 degrees of unsaturation (54:4 to 54:5), whereas CsDGAT2 may biosynthesize TAGs with 6 to 9 degrees of unsaturation (54:6 to 54:9). These data suggest that CsDGAT1 functions to increase the C18:1 and C18:2 content and that CsDGAT2 functions to increase the C18:3 content. These reports provide a good explanation for the increased C18:3 content in the *Csdgat1* KO camelina and the decreased C18:3 content in the *Csdgat2* KO camelina.

The EDD1 lines would retain the activities of CsDGAT2 and CsDGAT3 in regard to seed oil biosynthesis, and EDD2 lines would retain those of CsDGAT1 and CsDGAT3. *CsDGAT2* was more highly expressed in developing seeds compared to *CsPDAT1* (Supplementary Figure S4B,D), indicating that CsDGAT2 participates more in seed oil biosynthesis in the EDD1 lines, compensating for the absence of CsDGAT1 activity. However, given that the seed fatty acid composition of the EDD2 lines was unaltered, the activity of CsDGAT2 in the EDD1 lines is likely negligible. Therefore, it can be assumed that the seed fatty acid compositions of the EDD1 and EDD2 lines are dependent on the acyl preferences of CsDGAT3 (in the EDD1 lines) and of CsDGAT1 and CsDGAT3 (in the EDD2 lines), respectively. We predicted that in the EDD1 lines, the content of C18:3, which CsDGAT3 prefers, would be increased. Indeed, the EDD1 lines had an increased C18:3 content, but the C18:1 and C18:2 contents were also increased, which differed from the situation in the T_2_ seeds (Figure 2A and Figure 3C). Furthermore, the decreased C20:1 contents of the T_3_ seeds compared to the WT seeds suggested that CsDGAT1 prefers C20:1 as an acyl substrate (Figure 3C). The fact that the C20:1 contents in the EDD1 lines were decreased to a greater extent than those in the EDD2 lines [36] (Figure 3C) implies that CsDGAT1 contributes more to increasing the C20:1 content in seed oil than CsDGAT3 does. In the EDD2 lines, we expected to see a reduced C18:3 content because DGAT2 has a strong preference for C18:3 in plants [34,41]. However, there was only a slight alteration in the seed fatty acid composition, especially in the C18:3 content (Figure 4). This suggests that the activity of CsDGAT2 is much weaker than that of other CsDGATs, and only slightly affects fatty acid composition.

We observed reduced seed oil content and abnormal seed morphology in EDD2#9-2, #30-2, and #30-5 (Supplementary Figure S7). Initially, we thought that these phenotypes were the result of *CsDGAT2* KO. Given the low expression level of *CsDGAT2* (Supplementary Figure S4B) and the 1 to 3 missense mutations in their *CsDGAT2* homoeologs (Table 2), it was unclear whether the phenotypes were caused by mutation of *CsDGAT2*. Furthermore, the fatty acid composition and content of three lines were very different from those of the rest of the EDD2 lines, which have less difference in fatty acid composition and content among them (Figure 2B and Supplementary Figure S6A). As a result, we concluded that there was an unknown problem with these lines, and we could not exclude an off-target effect. This should be investigated further to resolve the discrepancy.

The germination rates of the EDD1, EDD2, and EDD3 lines were all 100% at 1 DAI, similar to that of the WT. Considering that the seed oil content and seed weight of EDD1 and EDD2 lines did not change (Figure 3A,B and Figure 4A,B), this germination rate seems reasonable. In addition, EDD3#1-5-7, which had an 18.8% lower seed oil content, had a germination rate identical to that of the WT. Other studies have reported that the germination rate of plants with reduced seed oil content was similar to that of the WT. The germination of AS11, the Arabidopsis *dgat1* mutant, which has a 25% reduced oil content [7,13], was delayed for 6 h compared to the WT, but its germination rate was similar to that of the WT [47].

In conclusion, we analyzed the biosynthetic functions and acyl preferences of CsDGAT1 and CsDGAT2, two seed-specific proteins in camelina, using *Csdgat1* and *Csdgat2* mutants generated by CRISPR/Cas9. Our results indicate that CsDGAT1 and CsDGAT2 participate in TAG biosynthesis, but their contributions are lower than that of CsDGAT3. Due to their functional redundancy in TAG biosynthesis, their exact contribution ratios were not determined, but the contributions of CsDGAT1 and CsDGAT2 were sufficient to affect the fatty acid composition. CsDGAT1 and CsDGAT2 prefer C20:1 and C18:3, respectively, as an acyl substrate. Collectively, these results showed that the activities of CsDGATs in camelina differ from those in Arabidopsis, although they have a very close phylogenetic relationship. These findings suggest that the overexpression or knockout of *CsDGAT1* or *CsDGAT2* could be applied to developing tailored oilseed plants, including camelina, with a high MUFA content or a high omega-3 fatty acid content in their seed oil. These findings, applying the diverse characteristics of camelina as an oilseed crop, along with gene editing techniques, are also poised to impact both agricultural and biotechnological industries, while enhancing the sustainability and efficiency of future agriculture. This study suggests that continuous exploration is warranted to fully harness the potential of camelina as a promising oilseed crop.

## 4. Materials and Methods

### 4.1. Biological Material

*Camelina sativa* cv. CAME was used as the wild-type parent for the mutagenesis of *CsDGAT1* and *CsDGAT2* genes. The camelina seeds were sown in soil at 4 °C for 2 days and then transferred to a culture room at 19 °C under long-day conditions (16-h light/8-h dark).

### 4.2. Subcellular Localization Analysis Using Nicotiana benthamiana Leaves

Each *CsDGAT2* homoeolog was fused to *yellow fluorescence protein* (*YFP*) lacking the stop codon at its 5′-end. The expression cassette was constructed from the cauliflower mosaic virus (CaMV) 35S promoter with the 5′-untranslated region leader (Ω) sequence of the tobacco mosaic virus and the 35S terminator using the Golden Gate cloning method [48]. The *monomeric red fluorescent protein* (*mRFP*)-fused *BrFAD2-1* and the tobacco bushy stunt virus p19 were used as an ER marker and a suppressor of virus-induced gene silencing, respectively [49,50]. After *Agrobacterium tumefaciens* (strain GV3101) was cultured at 28 °C overnight, the OD_600_ of Agrobacterium carrying *YFP*-fused *CsDGAT2*, *mRFP*-fused *BrFAD2-1*, and *p19* was adjusted to 0.8, 0.8, and 0.4, respectively, with an infiltration buffer [10 mM 2-(*N*-morpholino)ethanesulfonic acid (MES) (pH 5.6), 10 mM MgCl_2_, 200 μM acetosyringone]. Mixed Agrobacterium was infiltrated into 5-week-old *N. benthamiana* leaves. Agroinfiltrated *N. benthamiana* leaves were cut off and the fluorescence signal was checked using a TCS SP8 laser scanning confocal microscope (Leica, Germany) 36–48 h after infiltration. YFP and mRFP were excited at 514 nm and 561 nm, respectively, and their fluorescence was observed at 518–553 nm and 573–616 nm, respectively.

### 4.3. Reverse-Transcription Quantitative PCR Analysis

To determine the expression level of *CsDGAT1* and *CsDGAT2* homoeologs in camelina, total RNAs were extracted from cotyledons from 5-day-old seedlings, roots from 2-week-old seedlings, stems from 6-week-old plants, leaves from 6-week-old plants, flowers from 7-week-old plants, and developing seeds at stage 1 (DS1), DS2, DS3, DS4, and DS5 using a Spectrum^TM^ Plant Total RNA kit (Sigma-Aldrich, St. Louis, MO, USA). First-strand cDNAs were synthesized using 2 μg of total RNA and the RNA to cDNA EcoDry Premix (Clontech, Mountain View, CA, USA) at 42 °C for 40 min for reverse transcription. Primers for quantitative PCR (qPCR) were designed to specifically amplify each *CsDGAT1* and *CsDGAT2* homoeolog (Supplementary Table S6) using the method by Hayashi et al. [42], and the qPCR was conducted using 40 cycles of 94 °C for 30 s, 65 °C for 30 s, and 72 °C for 30 s, followed by 72 °C for 5 min, using a real-time PCR instrument StepOnePlus (Applied Biosystems, Waltham, MA, USA). *CsActin2* was used as a reference transcript [28].

### 4.4. Guide RNA Design and Vector Construction

The first exons of the *CsDGAT1A* homoeolog (GenBank accession No. XM_010417066.2) and *CsDGAT2A* homoeolog (GenBank accession No. XM_010505546.2) were used as the target sequences for genome editing. The Cas Designer tool (http://rgenome.net/cas-designer/, accessed on 24 June 2024) [43] was employed to select two sgRNAs that matched the sequences of the CsDGAT1 and CsDGAT2 homoeologs. These sgRNAs were chosen based on the following three criteria recommended by Cas Designer: (1) a GC content of 40–60%, (2) an out-of-frame score of over 67 points, and (3) no 1–3 nt mismatches. The information on sgRNAs for *CsDGAT1*s and *CsDGAT2*s is listed in Supplementary Table S2.

To induce a mutation in *CsDGAT1* and *CsDGAT2* homoeologs, we used pHEE401E-Red vector [36], which replaced the *hygromycin phosphotransferase* gene with the *DsRed* marker gene, containing EC1.2 promoter-driven *Zea mays* codon-optimized *Cas9* (*zCas9*) from *Streptococcus pyogenes* (enhancer:EC1.2P:*zCas9*). Two sgRNAs were inserted into the vector using the Golden Gate cloning method [36,51]. Briefly, the fragment containing two sgRNAs was twice amplified with different primer sets and pCBC-DT1T2 as a template. The destination vector and the fragment containing two sgRNAs were simultaneously digested with *Bsa*I and ligated together using T4 DNA ligase at 37 °C for 5 h, 50 °C for 5 min, and 80 °C for 10 min. The ligated clones were transformed into *Escherichia coli* DH5α and the binary vectors harboring two sgRNAs were selected using Sanger sequencing. The completed vectors are depicted in Supplementary Figure S5. The sequences of primers used in this procedure are listed in Supplementary Table S6.

### 4.5. Camelina Transformation and Selection of Transformants

*Agrobacterium tumefaciens* (GV3101 strain) cells were transformed with each constructed vector using the freeze–thaw method [52]. Camelina was transformed using a simple floral dip method two or three times at 1-week intervals [30,36,53]. In brief, Agrobacterium GV3101 harboring the binary vector described above was inoculated into 1 L of LB broth containing 50 μg/mL of kanamycin and 50 μg/mL of rifampicin and incubated at 28 °C for 24 h. Transformed seeds that emitted orange-red fluorescence were screened using a green LED and a red filter in the dark [29] and grown on soil under the conditions described in Section 4.1.

### 4.6. Homoeolog-Specific PCR and Sanger Sequencing–Based Genotyping

Genomic DNA from 4-week-old camelina leaf samples was extracted using a magnetic bead-type Genomic DNA Prep Kit for Plants (BIOFACT, Daejeon, Republic of Korea), following the manufacturer’s instructions. Briefly, about 100 mg of leaf tissue was sampled from three different leaves of one camelina plant and placed in a 2 mL microcentrifuge tube containing 2.3 mm chrome beads (BioSpec Products, Bartlesville, OK, USA). The leaf samples were frozen in liquid nitrogen and ground with a Tissue Lyser (Qiagen, Hilden, Germany). Lysis buffer was added to the 2 mL microcentrifuge tube. Following 13,000 rpm centrifugation at room temperature for 3 min, the supernatant was transferred to a 96-deep-well plate. Isopropanol and magnetic beads were combined and transferred to a 96-deep-well plate. Magnetic beads attached to nucleic acids, proteins, and so on, were separated with a 96 multi-magnet pipet and washed three times in a 96-well round-bottom plate, and the genomic DNA was eluted. Using this DNA as a template, homoeolog-specific PCRs were performed for each individual. PCR was conducted by using 30 cycles of 94 °C for 20 s, 54 °C for 20 s, and 72 °C for 30 s, followed by 72 °C for 5 min. The PCR products were purified with a magnetic bead-type PCR Purification Kit (BIOFACT, Republic of Korea) for Sanger sequencing, following the manufacturer’s instructions. The sequences of homoeolog-specific primers are listed in Supplementary Table S6.

The results (ab1 file) of the homoeolog-specific PCR-based Sanger sequencing from *Csdgat1* or *Csdgat2* KO camelina plants were analyzed with the web-based tools TIDE (http://shinyapps.datacurators.nl/tide/, accessed on 24 June 2024) [44] or DSDecodeM (http://skl.scau.edu.cn/dsdecode/, accessed on 24 June 2024) [45].

### 4.7. Fatty Acid Analysis

For rapid analysis of fatty acid composition, about 10 mg of camelina seeds were crushed in a 2 mL microcentrifuge tube containing two to three 2.3 mm chrome beads and 0.5 mL of *n*-hexane using a Tissue Lyser (Qiagen). After centrifugation, the supernatant was transferred to a new 2 mL microcentrifuge tube and mixed with 0.5 mL of 0.5 M sodium methoxide in methanol. Then, 1 mL of 0.9% (*w*/*v*) NaCl was added to the sample, and fatty acid methyl esters (FAMEs) in supernatant were transferred to vials for gas chromatograph (GC). To quantify the camelina seed oil, weighed seed samples were crushed and mixed with 0.5 mL of 5% (*v*/*v*) H_2_SO_4_ in methanol containing pentadecanoic acid (15:0) as an internal standard. Fatty acids were transmethylated at 85 °C for 90 min, and FAMEs were extracted three times with 1 mL of *n*-hexane after 1.5 mL of 0.9% (*w*/*v*) NaCl was added to the sample and mixed. The FAMEs were dried with nitrogen gas and dissolved in 0.2 mL *n*-hexane. The fatty acid composition and seed oil content were determined by a GC-2010 plus (Shimadzu, Kyoto, Japan) gas chromatograph with a flame ionization detector (FID) and a 30 m × 0.25 mm (inner diameter) HP-FFAP column (Agilent, Santa Clara, CA, USA), while the oven temperature was increased from 150 °C to 220 °C at 30 °C/min and then held constant for 11 min. Ultrapure nitrogen gas was used as the carrier gas.

### 4.8. Phylogenetic Analysis and Multiple Alignment

To generate the phylogenetic tree, the deduced amino sequences of *DGAT1* and *DGAT2* from various plant species were obtained through the Basic Local Alignment Search Tool (BLAST) at the National Center for Biotechnology Information (NCBI) (http://blast.ncbi.nlm.nih.gov/Blast.cgi, accessed on 24 June 2024). The deduced amino sequences of *CsDGAT1*s and *CsDGAT2*s were obtained from Kim et al. [11] and the camelina genome database in EnsemblPlants (https://plants.ensembl.org/Camelina_sativa/Info/Index, accessed on 24 June 2024), respectively. The phylogenetic tree was constructed using MEGA6 software (Ver. 6.06) (https://www.megasoftware.net, accessed on 24 June 2024) using the maximum-likelihood method. The deduced amino acid sequences of *DGAT1* and *DGAT2* were aligned using DNASTAR15 MegAlin software (Ver. 15.1.0) with Clustal W and the resultant multiple alignments were shown using GENEDOC software (Ver. 2.6.002).

### 4.9. Seed Phenotyping

Seed weight was determined by weighing 100 seeds in triplicate. The germination rate was determined as follows. Seeds were surface-sterilized with 70% (*v*/*v*) ethanol for 1 min, and subsequently with a 20% (*v*/*v*) concentration of commercial bleach and 0.01% (*v*/*v*) Tween-20 for 10 min. One hundred surface-sterilized seeds were placed on a 90 × 20 mm petri dish containing a half-strength MS agar medium without sucrose. After synchronization at 4 °C for 2 days, the seeds were transferred to a 25 °C growth chamber with a 16 h light/8 h dark photoperiod. Three independent plates were checked at 1 day to 5 days after transferring to a 25 °C growth chamber.

## Figures and Tables

**Table 1:** Patterns of indels, zygosity of three *CsDGAT1* homoeologs, and predicted genotypes of *Csdgat1* mutants (EDD1) generated by CRISPR/Cas9 system. D + number and I + number indicate the number of deleted and inserted nucleotides, respectively. A, B, and C in genotype represent *CsDGAT1A*, *CsDGAT1B*, and *CsDGAT1C* homoeologs, respectively. Lowercase letters and lowercase letters with ′ in genotypes show a nonsense mutation in *CsDGAT1* and a missense mutation in *CsDGAT1*, respectively. Biallele, biallelic mutant; Chi, chimeric mutant; Hom, homozygous mutant; Het, heterozygous mutant, ?, unidentified genotype.

Line No.	*CsDGAT1A*		*CsDGAT1B*		*CsDGAT1C*		Predicted Genotype
	Indel	Zygosity	Indel	Zygosity	Indel	Zygosity	
EDD1#10-1, -3, -4, -10	D6	Het	-	WT	-	WT	Aa′BBCC
EDD1#10-5	D39 + D6	Het	-	WT	-	WT	Aa′BBCC
EDD1#10-6, -7	D39 + D6	Hom	-	WT	-	WT	a′a′BBCC
EDD1#22-1, -4	D3 + D27, D121	Biallele	D121	Hom	D121	Hom	aa′bbcc
EDD1#22-2	D3 + D27, D121	Biallele	D121	Het	D121	Het	aa′BbCc
EDD1#22-3	D3 + D27	Hom	D121	Hom	D121	Het	a′a′bbCc
EDD1#22-5	D3 + D27	Hom	D121	Het	D121	Hom	a′a′Bbcc
EDD1#22-6, -7	D3 + D27, D121	Biallele	D121	Hom	D121	Het	aa′bbCc
EDD1#22-8	D3 + D27, D121	Biallele	D121	Het	D121	Hom	aa′Bbcc
EDD1#45-1, -3, -6	-	WT	-	WT	D14	Het	AABBCc
EDD1#45-8, -9	-	WT	-	WT	D14	Hom	AABBcc
EDD1#47-1, -2, -3, -8	I1	Hom	D121	Hom	D120	Hom	aabbc′c′
EDD1#47-4, -9, -10	Chi	-	D121	Hom	D120	Het	??bbCc′
EDD1#47-5, -6	Chi	-	D121	Hom	D120	Hom	??bbc′c′

**Table 2:** Patterns of indels, zygosity of three *CsDGAT2* homoeologs, and predicted genotypes of *Csdgat2* mutants (EDD2) generated by CRISPR/Cas9 system. D + number and I + number indicate the number of deleted and inserted nucleotides, respectively. A, B, and C in genotype represent *CsDGAT2A*, *CsDGAT2B*, and *CsDGAT2C* homoeologs, respectively. Lowercase letters and lowercase letters with ′ in genotypes show a nonsense mutation in *CsDGAT2* and a missense mutation in *CsDGAT2*, respectively. Biallele, biallelic mutant; Hom, homozygous mutant.

Line No.	*CsDGAT2A*		*CsDGAT2B*		*CsDGAT2C*		Predicted Genotype
	Indel	Zygosity	Indel	Zygosity	Indel	Zygosity	
EDD2#9-2, -4, -5 -8	D37	Hom	D2	Hom	D9	Hom	aabbc′c′
EDD2#9-3, -7, -9, -10	D37	Hom	D37, D2	Biallele	D9	Hom	aabbc′c′
EDD2#24-1	D9	Hom	D2	Hom	D10, D2	Biallele	a′a′bbcc
EDD2#24-2	D9	Hom	D45	Hom	D10	Hom	a′a′bbcc
EDD2#24-3, -4, -6, -8	D9	Hom	D45, D2	Biallele	D2	Hom	a′a′bbcc
EDD2#24-7	D9	Hom	D45	Hom	D10, D2	Biallele	a′a′bbcc
EDD2#24-9, -10	D9	Hom	D45, D2	Biallele	D10, D2	Biallele	a′a′bbcc
EDD2#25-1	D1 + D8	Hom	D5	Hom	D2	Hom	a′a′bbcc
EDD2#25-2, -5	D1 + D8	Hom	D5, D10	Biallele	I1	Hom	a′a′bbcc
EDD2#25-3, -9 -10	D1 + D8	Hom	D5, D10	Biallele	D2, I1	Biallele	a′a′bbcc
EDD2#25-6	D1 + D8	Hom	D10	Hom	D2, I1	Biallele	a′a′bbcc
EDD2#25-7, -8	D1 + D8	Hom	D5, D10	Biallele	D2	Hom	a′a′bbcc
EDD2#30-1, -2 -4, -6, -9	D36	Hom	D9, D10	Biallele	D38, D9	Biallele	a′a′b′bcc′
EDD2#30-3	D36	Hom	D9, D10	Biallele	D9	Hom	a′a′b′bc′c′
EDD2#30-5	D36	Hom	D10	Hom	D38	Hom	a′a′bbcc
EDD2#30-7	D36	Hom	D9	Hom	D38, D9	Biallele	a′a′b′b′cc′
EDD2#30-8	D36	Hom	D9, D10	Biallele	D38	Hom	a′a′b′bcc
EDD2#30-10	D36	Hom	D10	Hom	D38, D9	Biallele	a′a′bbcc′
EDD2#42-1, -2	D4	Hom	I3, D5	Biallele	D38, D10	Biallele	aab′bcc
EDD2#42-3, -5	D2, D4	Biallele	I3, D5	Biallele	D38, D10	Biallele	aab′bcc
EDD2#42-4	D2	Hom	I3, D5	Biallele	D38	Hom	aab′bcc
EDD2#42-6	D2, D4	Biallele	I3	Hom	D38, D10	Biallele	aab′b′cc
EDD2#42-7	D4	Hom	I3	Hom	D38, D10	Biallele	aab′b′cc
EDD2#42-8	D2, D4	Biallele	D5	Hom	D38, D10	Biallele	aabbcc
EDD2#42-9	D2	Hom	D5+I1	Biallele	D38	Hom	aabbcc
EDD2#42-10	D4	Hom	D5	Hom	D38, D10	Biallele	aabbcc
EDD2#44-1, -2, -3	D2, D9	Biallele	D1, I1	Biallele	D3	Hom	aa′bbc′c′
EDD2#44-4, -8	D2, D9	Biallele	D1	Hom	D3	Hom	aa′bbc′c′
EDD2#44-5, -7	D2	Hom	D1, I1	Biallele	D10, D3	Biallele	aabbcc′
EDD2#44-6	D9	Hom	D1, I1	Biallele	D10, D3	Biallele	a′a′bbcc′
EDD2#44-9	D2	Hom	D1, I1	Biallele	D3	Hom	aabbc′c′
EDD2#44-10	D2, D9	Biallele	D1, I1	Biallele	D10, D3	Biallele	aa′bbcc′
EDD2#59-2, -3, -4, -6	D4	Hom	D37, D10	Biallele	D10, D2	Biallele	aabbcc
EDD2#59-5	D4	Hom	D10	Hom	D10, D2	Biallele	aabbcc
EDD2#59-7, -10	D4	Hom	D10	Hom	D2	Hom	aabbcc
EDD2#59-8, -9	D4	Hom	D37, D10	Biallele	D2	Hom	aabbcc
EDD2#66-1, -7, -9	D2, D4	Biallele	D2	Hom	D2	Hom	aabbcc
EDD2#66-4, -6, -8	D4	Hom	D2	Hom	D2	Hom	aabbcc
EDD2#66-5, -10	D2	Hom	D2	Hom	D2	Hom	aabbcc
EDD2#137-2, -3, -4, -8, -9	D36	Hom	D2	Hom	D4 + I1, D41	Biallele	a′a′bbc′c
EDD2#137-5	D36	Hom	D2	Hom	D4 + I1	Hom	a′a′bbc′c′
EDD2#137-6, -7	D36	Hom	D2	Hom	D41	Hom	a′a′bbcc
EDD2#147-2	D9	Hom	D37, D10	Biallele	D39, D2	Biallele	a′a′bbc′c
EDD2#147-3, -7, -9	D9	Hom	D37, D10	Biallele	D39	Hom	a′a′bbc′c′
EDD2#147-4, -8	D9	Hom	D37	Hom	D39	Hom	a′a′bbc′c′
EDD2#147-5	D9	Hom	D37	Hom	D2	Hom	a′a′bbcc
EDD2#147-10	D9	Hom	D10	Hom	D39, D2	Biallele	a′a′bbc′c

## Data Availability

The data presented in this study are available on request from the corresponding author.

## Supplementary Materials

The following supporting information can be downloaded at: https://www.mdpi.com/article/10.3390/ijms25136944/s1.
