# Supplementary material for: Functional Characterization of the Effects of CsDGAT1 and CsDGAT2 on Fatty Acid Composition in Camelina sativa
Source: Int J Mol Sci. 2024 Jun 25;25(13):6944. doi: 10.3390/ijms25136944 (PMC11240937; doi:10.3390/ijms25136944)
Supplement: Supplementary file 1 [file ijms-25-06944-s001.zip › ijms-3017094-supplementary.pdf]

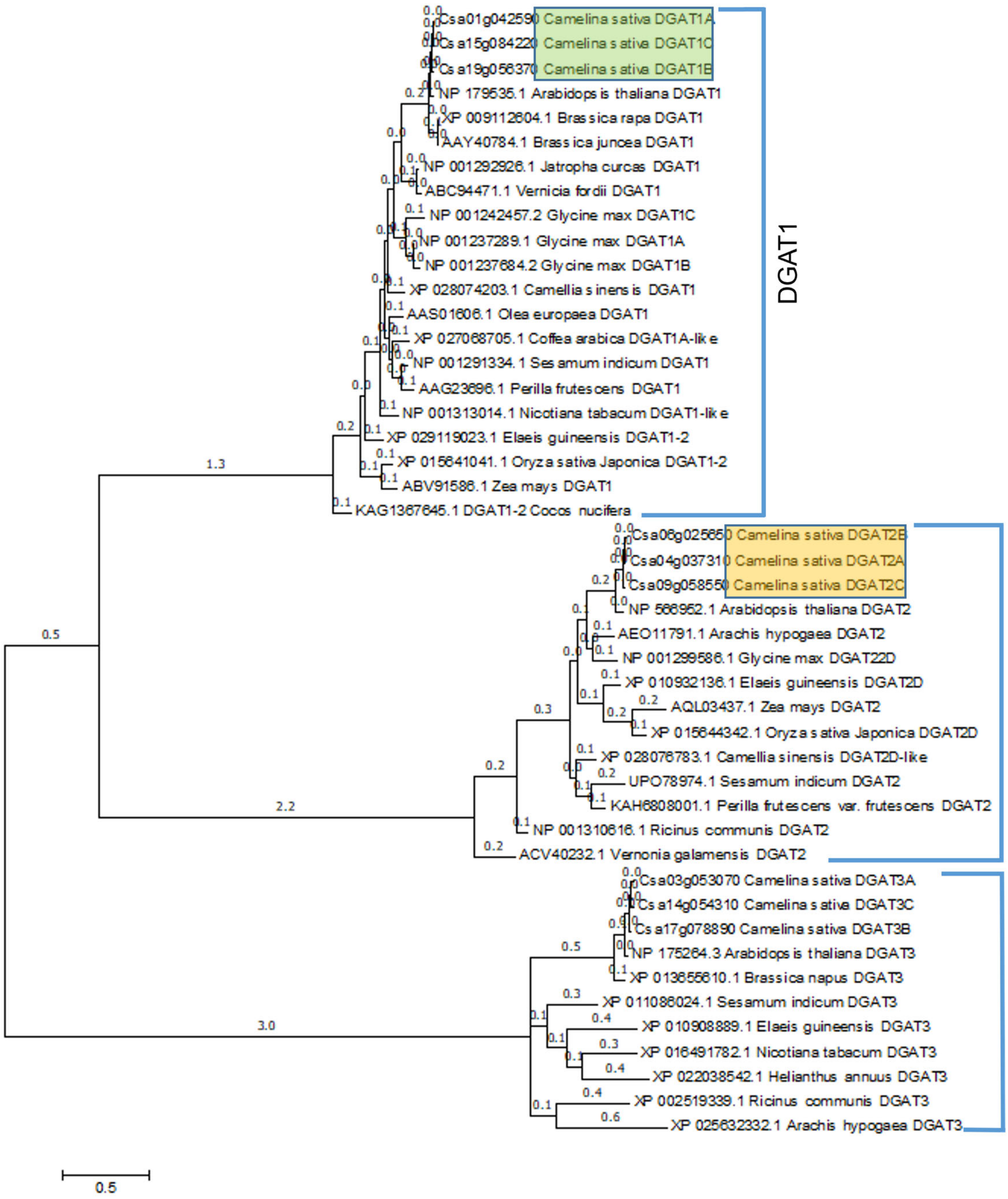

Figure S1. Phylogenetic relationship among DGAT1s and DGAT2s in plants. The phylogenetic relationship was assessed with MEGA6 using the maximum-likelihood method. The tree is drawn to scale, with branch lengths corresponding to the number of substitutions per site (listed above the branches). All positions containing gaps and missing data were omitted. Numbers in front of the scientific name of plant species and DGAT represent GenBank accession numbers. In the case of camelina DGATs, locus IDs from EnsemblPlants are presented.

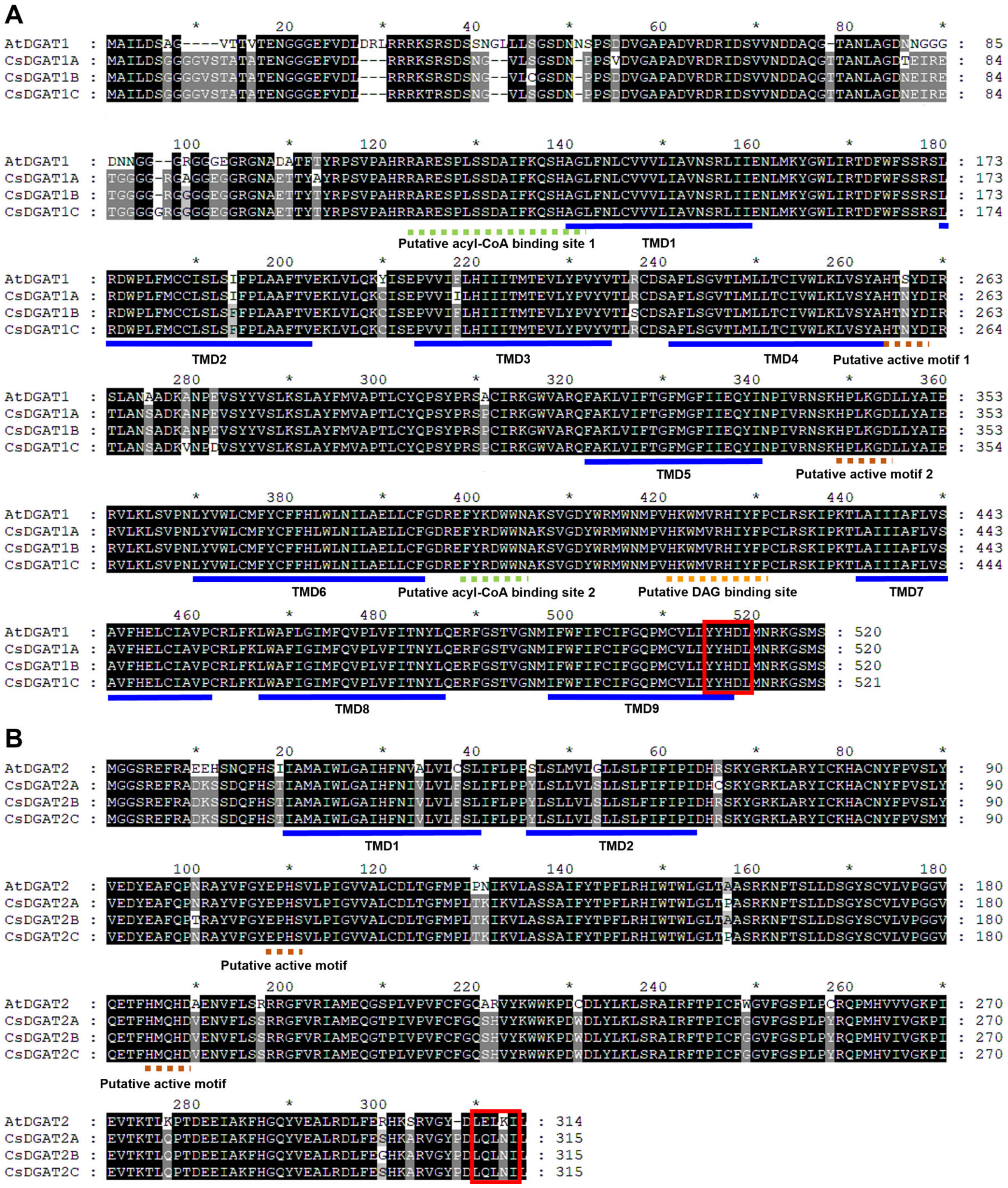

Figure S2. Multiple sequence alignment of CsDGAT1s and CsDGAT2s. (A) Multiple sequence alignment of CsDGAT1s with AtDGAT1. (B) Multiple sequence alignment of CsDGAT2s with AtDGAT2. Amino acid sequences shaded in black and gray indicate completely identical and three-quarters identical residues, respectively. Blue lines indicate transmembrane domains and red boxes represent ER retrieval motifs.

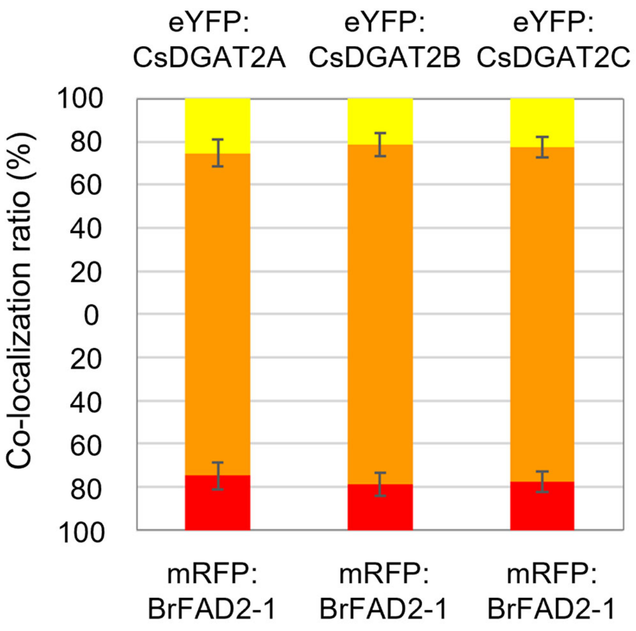

Figure S3. Quantification of co-localized eYFP and mRFP fluorescence. The percentage of signal intensity shown in the co-localized pixels (middle; orange) relative to total signal was measured for both the yellow (top; eYFP:CsDGAT2A, eYFP:CsDGAT2B, and eYFP:CsDGAT2C) and red (bottom; mRFP:BrFAD2-1) values. Yellow and red values denote the difference between 100% and the overlap measured for the corresponding co-localized pixels. The extent of co-localization signal intensity is quantified in the full images shown in Figure 1 using Pearson's correlation coefficient.

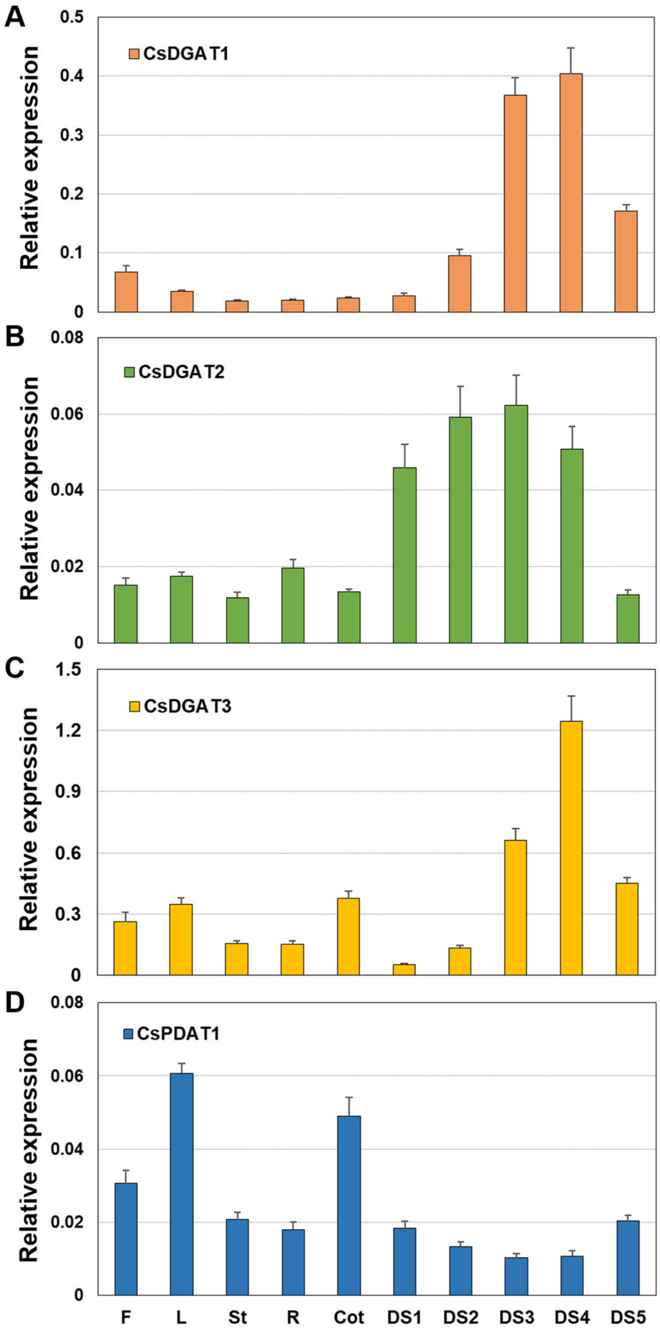

Figure S4. Transcript levels of *CsDGAT*s and *CsPDAT1*s using RT-qPCR with the primer set for simultaneous amplification of three homoeologs. (A) to (D) Relative expression of *CsDGAT1*s, *CsDGAT2*s, *CsDGAT3*s, and *CsPDAT1*s in flowers (F), leaves (L), stems (St), roots (R), cotyledons (Cot), and each stage of developing seeds [developing seed stage 1 (DS1), DS2, DS3, DS4, and DS5]. The experiment was performed in technical triplicate and error bar indicates standard deviation.

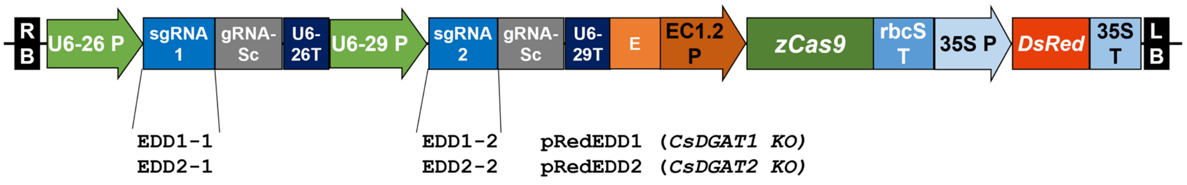

Figure S5. Binary vector constructs for *CsDGAT1* and *CsDGAT2* gene editing. The pRedEDD1 and pRedEDD2 vectors containing sgRNA1 and sgRNA2 were used to generate *CsDGAT1* and *CsDGAT2* KO camelina plants, respectively. Nucleotide sequences, which correspond to sgRNA1 and sgRNA2, are shown. U6-26P and U6-29P, U6 promoters from *Arabidopsis thaliana*; U6-26T and U6-29 T, U6 terminators from *A. thaliana*; E, enhancer; EC1.2P, egg cell 1.2 promoter; *zCas9*, *Zea mays* codon-optimized *Cas9* from *Streptococcus pyogenes*; rbcS T, the terminator of ribulose-1,5-bisphosphate carboxylase/oxygenase (rubisco) small subunit from pea (*Pisum sativum*); 35S P, CaMV 35S promoter; 35S T, CaMV 35S terminator; RB, right border; LB, left border.

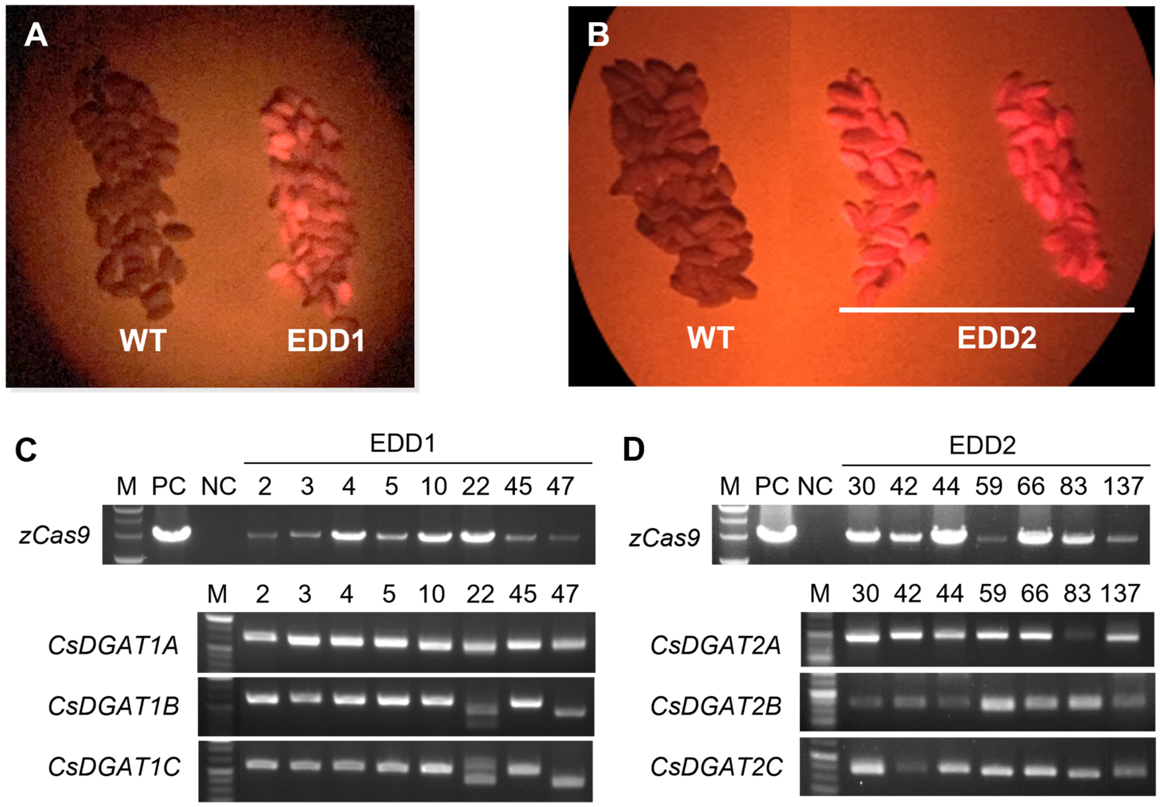

Figure S6. Selection of transgenic camelina seeds with DsRed fluorescence and homoeolog-specific PCR. (A,B) Screening of EDD1 (A) and EDD2 (B) seeds showing orange-red fluorescence. (C) *zCas9*- and *CsDGAT1* homoeolog-specific PCR in EDD1 mutants. (D) *zCas9*- and *CsDGAT2* homoeolog-specific PCR in EDD2 mutants. In (C) and (D), numbers above the gel image indicate the line number. M, marker; PC, positive control [pRedEDD1 and pRedEDD2 vectors in (C) and (D), respectively]; NC, negative control (WT camelina).

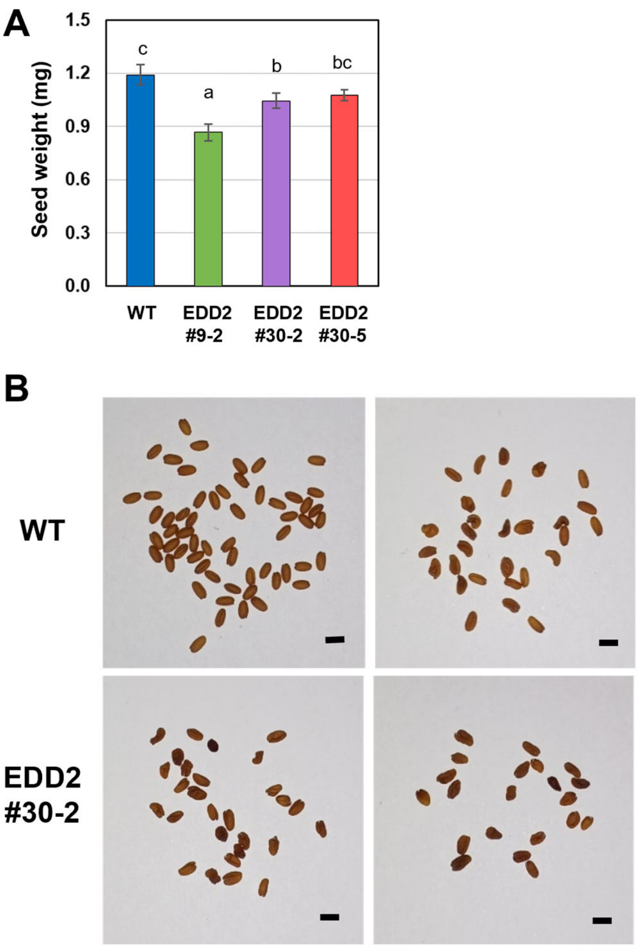

Figure S7. Seed phenotypes of EDD2#9-2, #30-2, and #30-5. (A) Seed weights of EDD2#9-2, #30-2, and #30-5 and WT. (B) Abnormal seed morphology was observed in the EDD2#9-2, #30-2, and #30-5 lines. Scale bars, 2 mm. The values were statistically analyzed using one-way ANOVA followed by Tukey's test ($P < 0.05$).

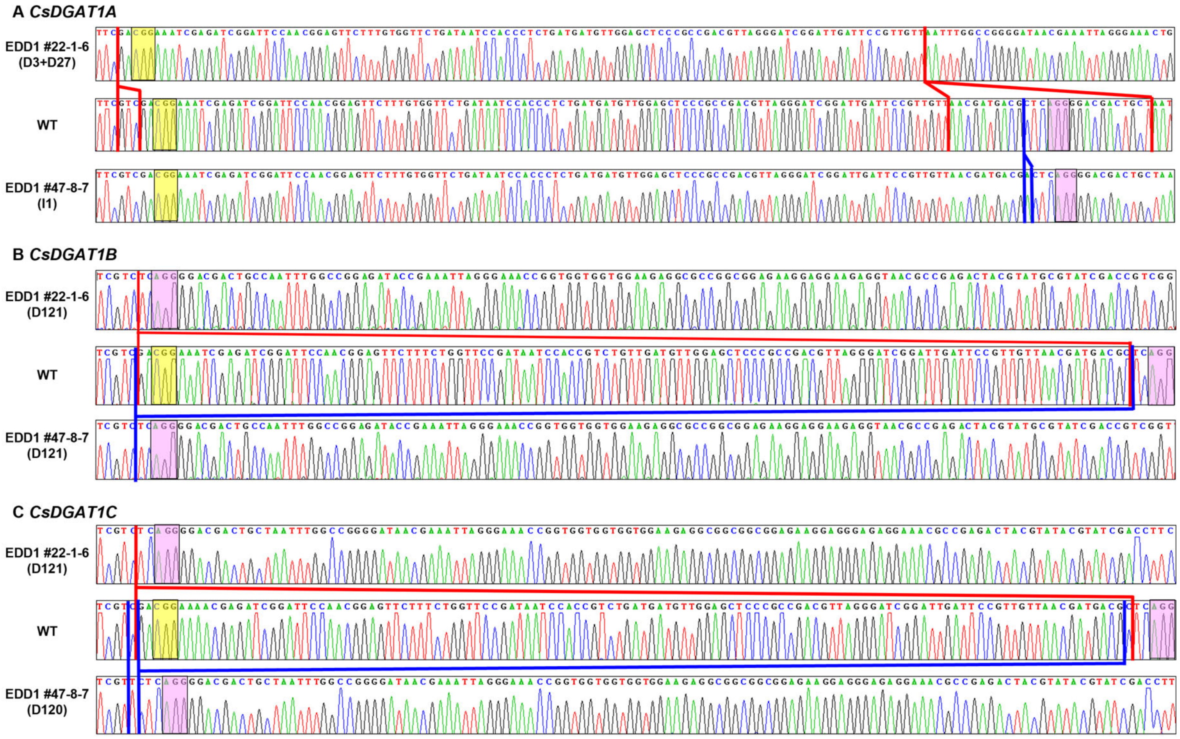

Figure S8. Mutation patterns of *CsDGAT1* homoeologs in EDD1 mutants, identified by Sanger sequencing. Shown are the altered nucleotide sequences of (A) *CsDGAT1A*, (B) *CsDGAT1B*, and (C) *CsDGAT1C* in EDD1#22-1-6 and #47-8-7. Yellow and pink boxes indicate the protospacer-adjacent motif (PAM) sequence of sgRNA1 and sgRNA2, respectively. Red and blue lines represent the indel or base substitution of EDD1#22-1-6 and #47-8-7, respectively.

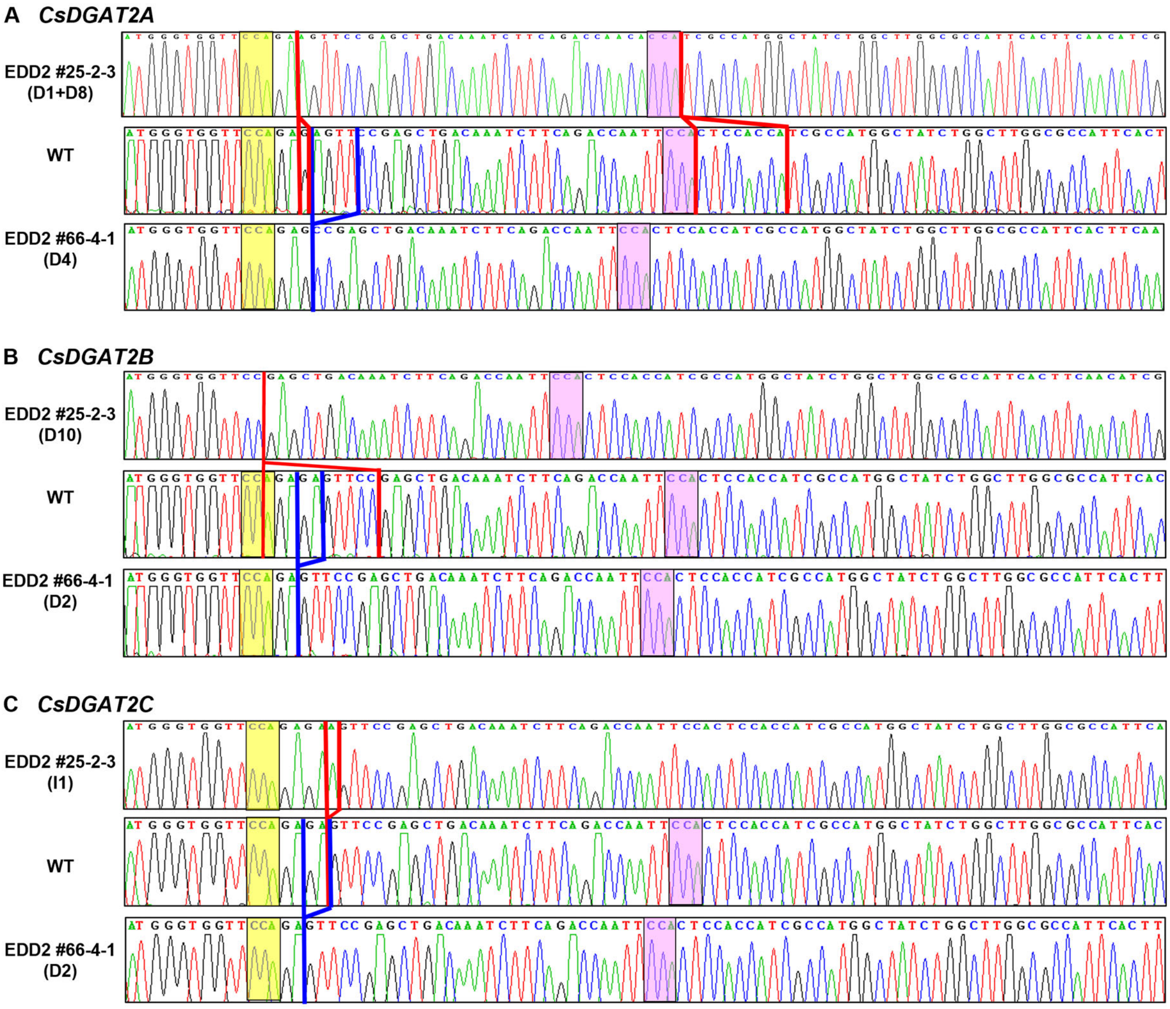

Figure S9. Mutation patterns of *CsDGAT2* homoeologs in EDD2 mutants, identified by Sanger sequencing. Shown are the altered nucleotide sequences of (A) *CsDGAT2A*, (B) *CsDGAT2B*, and (C) *CsDGAT2C* in EDD2#25-2-3 and #66-4-1. Yellow and pink boxes indicate the protospacer-adjacent motif (PAM) sequence of sgRNA1 and sgRNA2, respectively. Red and blue lines represent the deletion of EDD2#25-2-3 and #66-4-1, respectively.

Table S1. Information on *CsDGAT1* and *CsDGAT2*. Locus number was obtained from the camelina genome database in EnsemblPlants (https://plants.ensembl.org/camelina_sativa/info/index).

**Homoeolog**	**Locus number**	**Size**		Exon	transmembrane domain	Homology with AtDGAT1 or AtDGAT2 (%)
		**nt**	**aa**			
CsDGAT1A	Csa19g056370	1563	520	16	9	93.6
CsDGAT1B	Csa01g042590	1563	520	16	9	93.8
CsDGAT1C	Csa15g084220	1566	521	16	9	93.6
CsDGAT2A	Csa04g037310	948	315	9	2	90.1
CsDGAT2B	Csa06g025650	948	315	9	2	90.4
CsDGAT2C	Csa09g058550	948	315	9	2	90.1

Table S2. sgRNAs used for *CsDGAT1* and *CsDGAT2*. The underlined sequences at the 3′ ends indicate the protospacer-adjacent motif (PAM) sequence of each.

sgRNA	Sequence (5' to 3')	Position	Direction	GC Contents (w/o PAM)	Out of frame Score
EDD1-1	GTTTGTGGATCTTCGTCGACGG	69	+	47.4	73.9
EDD1-2	GTTGTTAACGATGACGCTCAGG	190	+	47.4	70.3
EDD2-1	TTGTCAGCTCGGAACTCTCTGG	11	-	52.6	68.4
EDD2-2	AGCCATGGCGATGGTGGAGTGG	48	-	63.2	81.0

Table S3. Proximate transformation efficiency of EDD1 and EDD2 camelina plants. 100-seed weight was measured in triplicate.

Line	Total T_1 seed weight (A)	Average 100-seed weight (B)	Calculated seed number (C=A/B*100*1,000)	Putative transgenic seeds (D)	Proximate transformation efficiency (D/C*100)
EDD1	29.58 g	109.7 mg	26,964	67	0.25%
EDD2	50.29 g	106.9 mg	47,044	172	0.37%

Table S4. Indel type and total indel frequency of EDD1 T_1 lines. The lines with total indel frequency over 25% are shown. The indel type with the highest frequency is represented in the indel column. D+number indicates the number of deleted nucleotides. Chi, chimera without outstanding indel pattern but with over 15% of total indel frequency; ND, not determined.

Line No.	*CsDGAT1A*		*CsDGAT1B*		*CsDGAT1C*	
	Indel	Total frequency (%)	Indel	Total frequency (%)	Indel	Total frequency (%)
EDD1#10	D6	36.4	-	-	-	-
EDD1#22	Chi	34.6	Chi	15.6	Chi	37.4
EDD1#45	-	-	-	-	D14	46.5
EDD1#47	D11	29.1	Chi	17.4	ND	ND
EDD1#49	-	-	-	-	D2	61.4

Table S5. Indel type and total indel frequency of EDD2 T_1 lines. The lines with total indel frequency over 25% are shown. The indel type with the highest frequency is represented in the indel column. D+number and I+number indicate the number of deleted and inserted nucleotides, respectively. Chi, chimera without outstanding indel pattern but with over 15% of total indel frequency; ND, not determined.

Line No.	*CsDGAT2A*		*CsDGAT2B*		*CsDGAT2C*	
	Indel	Total frequency (%)	Indel	Total frequency (%)	Indel	Total frequency (%)
EDD2#8	Chi	54.3	-	-	-	-
EDD2#9	D37	93.7	D37, D2	78.3	D9	98.0
EDD2#11	D10	95.8	Chi	51.9	D7	36.6
EDD2#17	Chi	14.7	D1, Chi	93.8	Chi	48.9
EDD2#24	D9	96.6	Chi	14.2	D2, D10	88.9
EDD2#25	D9	97.0	D5, D10	94.7	I1, D2	91.9

EDD2#26	Chi	47.8	D2, D1	91.5	D11, Chi	82.0
EDD2#27	D2, D44	75.5	D35, D8	91.5	-	-
EDD2#30	D36	96.5	D9, D10	95.8	Chi	52.1
EDD2#32	-	-	D19, D15	95.4	D3	42.5
EDD2#34	Chi	60.5	-	-	-	-
EDD2#38	-	-	Chi	45.1	D2	76.0
EDD2#42	D2, D4	93.3	I3, D5	88.0	Chi	56.9
EDD2#44	D9, D2	86.4	Chi	63.6	Chi	38.5
EDD2#54	Chi	73.9	D39, Chi	90.2	Chi	54.2
EDD2#59	D4, D2	96.1	Chi	58.5	D2, D10	88.3
EDD2#65	Chi	62.8	D2	91.6	Chi	44.2
EDD2#66	D2, D4	95.5	D2	96.0	D2	98.2
EDD2#71	-	-	-	-	D8, Chi	86.4
EDD2#74	I28	37.6	D2, Chi	61.3	D9	92.6
EDD2#80	Chi	39.3	-	-	-	-
EDD2#83	D36	84.9	D9	38.9	D24	97.7
EDD2#85	D2, D8	84.0	-	-	D36	85.3
EDD2#86	Chi	44.3	Chi	40.6	D9	93.5
EDD2#88	Chi	47.4	-	-	-	-
EDD2#91	Chi	71.2	-	-	-	-
EDD2#106	D10	40.8	D3	45.6	D8, D2	92.3
EDD2#114	D1	93.0	D37, D3	87.1	Chi	35.6
EDD2#115	I1	48.2	-	-	D7	48.2
EDD2#119	D36	47.2	-	-	-	-
EDD2#132	D9	89.3	D26	80.4	-	-
EDD2#137	D36	91.9	D2	81.7	Chi	20.7
EDD2#147	D9	95.2	D37, D10	83.9	Chi	45.7
EDD2#160	D4	42.0	-	-	-	-

Table S6. Sequences of primers used in this study.

Primer name	Primer sequence (5′ to 3′)	Purpose
YFP-CsDGAT2s fusion F	TTGGTCTCAAATGGGTGGTTCCAGAGAGTT	
YFP-CsDGAT2A fusion R	TTGGTCTCAAAGCTCAAAGAATGTTCAATTGAAGATC	Vector construction for subcellular localization assay of CsDGAT2s
YFP-CsDGAT2BC fusion R	TTGGTCTCAAAGCTCAAAGAATGTTCAGTTGAAGATC	
CsDGAT1DT1-F0	TGGTTTGTGGATCTTCGTCGAGTTTTAGAGCTAGAAATAGC	
CsDGAT1DT2-R0	AACGAGCGTCATCGTTAACAACCAATCTCTTAGTCGACTCTAC	Insertion of sgRNA for *CsDGAT1* KO to binary vector carrying *Cas9*
CsDGAT1DT1-BsF	ATATATGGTCTCGATTGGTTTGTGGATCTTCGTCGAGTT	
CsDGAT1DT2-BsR	ATTATTGGTCTCGAAACGAGCGTCATCGTTAACAACCAA	
CsDGAT2DT1-F0	TGGTTGTCAGCTCGGAACTCTCGTTTTAGAGCTAGAAATAGC	
CsDGAT2DT2-R0	AACCTCCACCATCGCCATGGCTCCAATCTCTTAGTCGACTCTAC	Insertion of sgRNA for *CsDGAT2* KO to binary vector carrying *Cas9*
CsDGAT2DT1-BsF	ATATATGGTCTCGATTGGTTGTCAGCTCGGAACTCTCGTT	

CsDGAT2DT2-BsR	ATTATTGGTCTCGAAACCTCCACCATCGCCATGGCTC CAA	
CsDGAT1A F	CTCTCGTGAATCTTTTTTCAA	*CsDGAT1A*-specific PCR for genotyping
CsDGAT1A R	CACCAAATTCCAAGATTGTTA	
CsDGAT1B F	ACAAACAAACCTTCTCTTAATATG	*CsDGAT1B*-specific PCR for genotyping
CsDGAT1B R	TTCGAAGACTGTTAAAGTATTTATT	
CsDGAT1C F	CTCAATATTCTCTCTTCATTGG	*CsDGAT1C*-specific PCR for genotyping
CsDGAT1C R	CTGTTAAAGTATCTGAAGAGATAA	
CsDGAT2A F	CGAGAAAGGATGAATCTTCT	*CsDGAT2A*-specific PCR for genotyping
CsDGAT2A R	CTTACGACCGTATTTCCTACA	
CsDGAT2B F	GGGTGGGTGGTTCGTTAA	*CsDGAT2B*-specific PCR for genotyping
CsDGAT2B R	CCTGAAAACAGAGGATTTTCAG	
CsDGAT2C F	TCTGAGTAGAATCTTCGATTCAT	*CsDGAT2C*-specific PCR for genotyping
CsDGAT2C R	CGGCCGTATTTGCTACGA	
CsDGAT1A qF	ACGGAGTTCTTTGTGGATCT	*CsDGAT1A*-specific RT-qPCR
CsDGAT1A qR	CCTCTTCCACCTCCCGCA	
CsDGAT1B qF	CCAATTTGGCCGGAGTTAC	*CsDGAT1B*-specific RT-qPCR
CsDGAT1B qR	TTACAGCAATAAGAACTACGACG	
CsDGAT1C qF	GTTGTCATCTTTCTTCATATTATTATAACT	*CsDGAT1C*-specific RT-qPCR
CsDGAT1C qR	GCTGAATTGGCTAGGGTC	
CsDGAT2A qF	ATCTTCATCCCAATCGATGTCT	*CsDGAT2A*-specific RT-qPCR
CsDGAT2A qR	CAACAACTCCAATCGGTAATTCT	
CsDGAT2B qF	CCACATTCGGTGTTACGTATC	*CsDGAT2B*-specific RT-qPCR
CsDGAT2B qR	CCAGGTACAAGAACAGAACTATAT	
CsDGAT2C qF	CTCCTGGTCTTAAGCTTTCTG	*CsDGAT2C*-specific RT-qPCR
CsDGAT2C qR	GTGGTTCATAACCAAAGACCTAT	
